# 3D intratumoral heterogeneity-based quantitative score from chest CT for preoperative prediction of visceral pleural invasion in lung adenocarcinoma: a multicenter study

**DOI:** 10.3389/fonc.2026.1837845

**Published:** 2026-05-12

**Authors:** Qunzhi Ouyang, Yanping Wu, Liuhan Zhou, Wanyin Qi, Sanhong Zhang, Yan Zhao, Jingyi Zuo

**Affiliations:** 1 Department of Radiology, Ningyuan County People’s Hospital, Yongzhou, Hunan, China; 2 Department of Radiology, Xiangtan Central Hospital, Xiangtan, Hunan, China; 3 Department of Radiology, The Affiliated Hospital of Southwest Medical University, Luzhou, Sichuan, China; 4 Department of Radiology, Liuyang Traditional Chinese Medicine Hospital, Changsha, Hunan, China; 5 Department of Radiology, The Fifth People’s Hospital of Xiangtan City, Xiangtan, Hunan, China; 6 School of Medicine and Life Sciences, Zhangjiajie College, Zhangjiajie, Hunan, China

**Keywords:** 3D ITH score, lung adenocarcinoma, multicenter study, stacking ensemble learning, visceral pleural invasion

## Abstract

**Introduction:**

Visceral pleural invasion (VPI) is a critical adverse prognostic factor in lung adenocarcinoma (LUAD). This study aimed to develop a stacking ensemble model that integrates three-dimensional intratumoral heterogeneity (3D ITH) scores with clinicoradiologic features to achieve accurate preoperative prediction of VPI in LUAD.

**Methods:**

This multicenter retrospective study included 1,301 patients with LUAD from three medical centers. Patients from Centers 1 and 2 were assigned to the development cohort, whereas those from Center 3 constituted the fixed external validation cohort. To calculate the 3D ITH score, we integrated local radiomic descriptors with global pixel distribution characteristics derived from whole tumor CT volumes. Clinicoradiologic features and 3D ITH scores were then used to construct six base machine learning models and a final stacking ensemble classifier. Model performance was primarily assessed using receiver operating characteristic analysis and the area under the curve (AUC). SHapley Additive exPlanations (SHAP) were used to quantify feature contributions and to interpret the final model.

**Results:**

The stacking ensemble classifier achieved the highest AUC for preoperative prediction of VPI in LUAD (AUC = 0.878), whereas XGBoost showed competitive performance on several threshold dependent metrics. SHAP analysis identified the 3D ITH score as the most influential predictor, followed by nodule size and CT density. Comparative experiments further showed that the stacking ensemble model outperformed the conventional radiomics signature (AUC = 0.841) and the clinicoradiologic comparative model (AUC = 0.776).

**Conclusion:**

The model integrating 3D ITH scores with clinicoradiologic features showed strong discrimination for preoperative prediction of VPI in LUAD. This approach may serve as a useful adjunct for preoperative risk stratification and individualized treatment planning.

## Introduction

Lung adenocarcinoma (LUAD), which accounts for 60% to 70% of non small cell lung cancer cases, is the most prevalent histological subtype of primary lung cancer worldwide and continues to impose a substantial burden on global health care systems (1, 2). Visceral pleural invasion (VPI), a critical adverse prognostic factor in LUAD, is closely associated with increased risks of postoperative recurrence, lymph node metastasis, and reduced overall survival, and it therefore plays an important role in tumor staging, treatment planning, and prognostic assessment (3–5). According to the International Association for the Study of Lung Cancer, tumors 3 cm or smaller with VPI should be upstaged from T1 to T2 and from stage IA to stage IB, which directly influences surgical planning (6). For example, patients with LUAD and VPI may derive greater benefit from lobectomy with systematic lymph node dissection than from sublobectomy, which has been identified as an independent risk factor for recurrence in this subgroup (7). At present, the reference standard for diagnosing VPI is pathological examination of surgical specimens. However, this approach is invasive, may be associated with complications such as pneumothorax and hemorrhage, and may not fully represent the pathological characteristics of the entire tumor (8, 9). Therefore, a noninvasive and accurate preoperative method for VPI assessment is urgently needed.

Radiomics can be regarded as a pixel level imaging surrogate of tumor expression patterns and molecular characteristics (10, 11), and it has demonstrated substantial value in characterizing the biological behavior of multiple tumor types (12–14). Conventional computed tomography (CT) has been widely used for screening and evaluating LUAD. Previous studies have shown that imaging features such as nodule density, nodule size, pleural indentation, and the pattern of nodule pleura interaction may provide useful clues for predicting VPI (15). However, the predictive performance of these visually assessed and inherently subjective CT features remains limited, with sensitivity and specificity insufficient for routine clinical use (16). Radiomics has emerged as a powerful complementary approach by extracting high dimensional quantitative features from medical images that are imperceptible to the human eye. This enables assessment of intratumoral heterogeneity and subtle pathological alterations, thereby improving the accuracy of preoperative VPI prediction in LUAD (17). Although both two dimensional and three dimensional radiomics models have shown promising performance, conventional radiomics generally treats the tumor as a relatively homogeneous whole and may fail to capture biologically meaningful spatial variation among intratumoral subregions (18–20). These limitations highlight the need for more advanced strategies that can characterize intratumoral heterogeneity (ITH) more comprehensively. Unlike conventional whole lesion radiomic descriptors such as entropy, which summarize overall intensity or texture heterogeneity, the ITH framework identifies spatially organized intratumoral subregions based on local radiomic patterns and quantifies their spatial distribution, thereby capturing not only the magnitude but also the organization of heterogeneity.

To address these challenges, Li et al. (21) proposed a dimensionless ITH score that integrates global and local pixel information to quantify intratumoral heterogeneity and demonstrated performance advantages over conventional radiomics in tissue subtype classification (22) and pathological invasiveness prediction (23–25). Nevertheless, most existing ITH scores are derived from two dimensional representations of the largest tumor cross section and may therefore fail to reflect the full three dimensional complexity of the lesion. In addition, prior studies have primarily focused on ground glass nodules (22–25), which limit generalizability to a broader spectrum of lung nodules. To overcome these limitations, Zuo et al. (26) recently introduced a 3D ITH score that synthesizes local radiomic features with global pixel distribution patterns extracted from the entire tumor volume on volumetric CT. In contrast, the corresponding 2D ITH score is derived from a single maximum slice. By incorporating the full spatial heterogeneity of the lesion, the 3D ITH score provides a more comprehensive and robust characterization of tumor biology in LUAD.

Building on these developments, both 2D and 3D ITH scores have shown promise for quantifying intratumoral heterogeneity and predicting tumor biological behavior. However, their value for preoperative prediction of VPI remains insufficiently studied (21–26). To address this gap, we conducted a multicenter study integrating 2D and 3D ITH scores with clinicoradiologic features to develop a machine learning based model for VPI prediction in LUAD. Using a large cohort of 1,301 patients from three independent medical centers, we aimed to improve the accuracy and robustness of preoperative VPI assessment and to provide a reliable noninvasive tool to support surgical planning and individualized treatment.

## Materials and methods

### Multicenter CT dataset acquisition

This multicenter retrospective cohort study included patients with LUAD who underwent curative surgical resection at three tertiary hospitals, Xiangtan Central Hospital, the Affiliated Hospital of Southwest Medical University, and Liuyang Traditional Chinese Medicine Hospital, between January 2018 and January 2024. In accordance with institutional protocols, all patients underwent preoperative non contrast chest CT using a standardized thin section protocol with a slice thickness of 1.5 mm or less. The acquisition parameters were consistent with those reported in previous studies and were harmonized across participating centers (25, 26).

Eligible patients met the following inclusion criteria: (1) histopathological confirmation of LUAD; (2) availability of high-resolution preoperative CT images; and (3) no evidence of distant metastasis at diagnosis. Exclusion criteria included: (1) histologically confirmed metastatic disease or multiple primary malignancies; (2) CT images of insufficient quality for reliable analysis; and (3) prior receipt of neoadjuvant therapy or radiotherapy.

### Compliance with ethical requirements

This study was approved by the institutional review boards of all participating centers. The approval numbers were as follows: the Affiliated Hospital of Southwest Medical University, KY2020147; Liuyang Traditional Chinese Medicine Hospital, 2022-016; and Xiangtan Central Hospital, 2021-07-009. Because of the retrospective design, the requirement for informed consent was waived by the respective institutional review boards.

### CT lesion mask segmentation

CT lesion masks were independently segmented by two board-certified radiologists with more than 10 years of experience in thoracic imaging. Both radiologists were blinded to pathological outcomes. Segmentation was performed using ITK-SNAP (Version 4.13.0), a widely validated medical image segmentation tool.

Radiologists manually delineated tumor boundaries on each axial CT slice (slice thickness ≤1.5 mm), including both solid components and ground-glass opacities, while excluding adjacent normal lung parenchyma, vessels, bronchi, and pleural structures. The segmentation spanned from the cranial to the caudal extent of the lesion to ensure complete tumor coverage without inclusion of non-tumor tissues.

### CT radiological feature evaluation

Radiological features were independently evaluated by two board certified thoracic radiologists, each with more than 10 years of experience, who were blinded to pathological outcomes. The evaluated features included lesion location, boundary clarity, well defined or ill defined; lobulation; spiculation; vascular convergence; vacuole sign; pleural indentation; lesion shape, regular or irregular; and CT density type, pure ground glass nodule, subsolid nodule, or solid nodule.

In cases of disagreement, a consensus meeting was held to resolve discrepancies and ensure consistent and reliable radiological feature interpretation.

### ITH score calculation

#### Overall strategy

**Figure 1:**
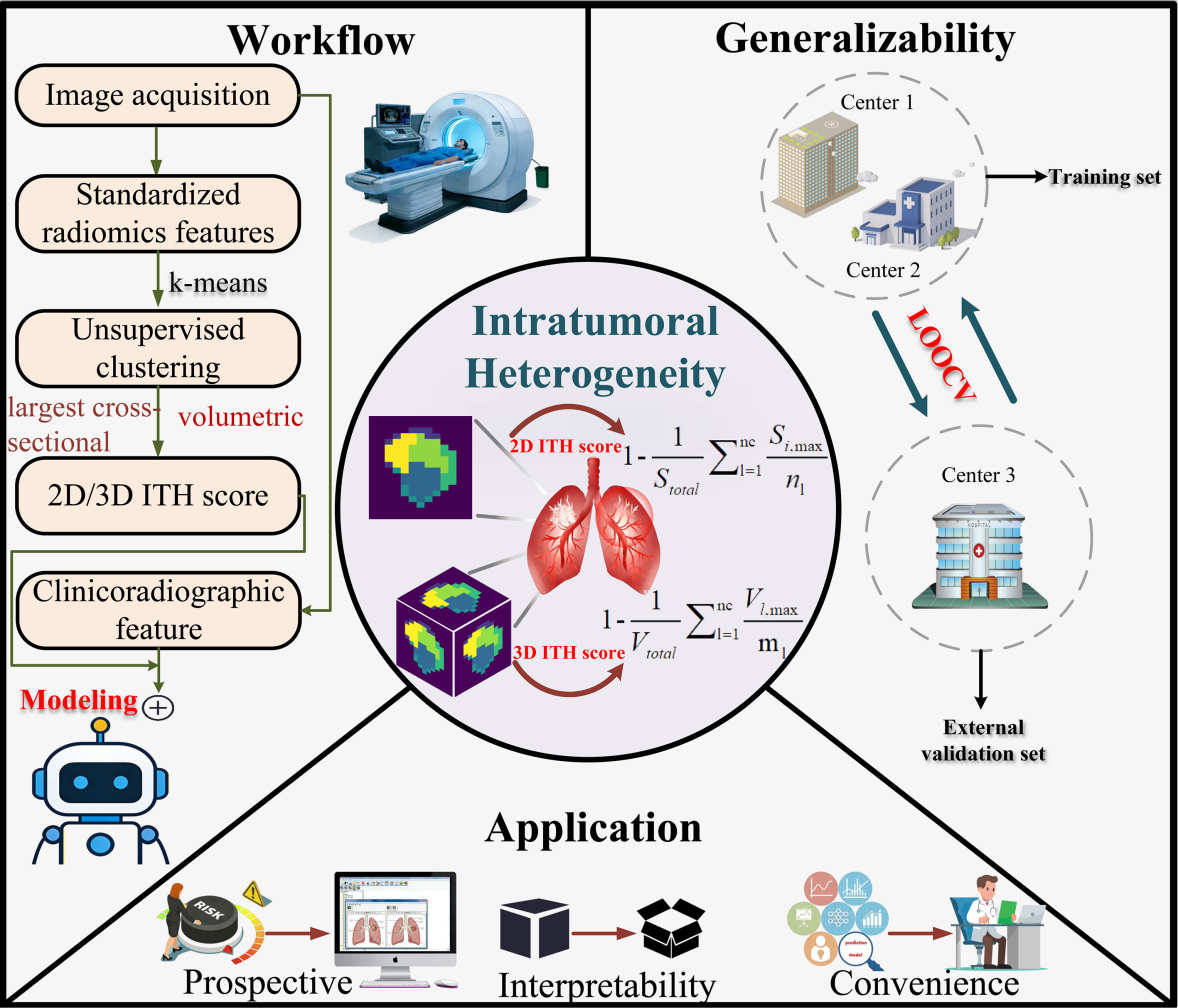
Schematic diagram of the experimental design framework in this study.

A systematic workflow, shown in **Figure 1**, comprised three interconnected modules: local radiomics feature extraction, global pixel clustering, and calculation of the 2D and 3D ITH scores. First, standardized local radiomic descriptors were extracted within spatially defined windows to capture fine scale tissue characteristics. According to the public ITHscore implementation, these descriptors were derived from the original PyRadiomics feature classes, including first order, shape based, gray level co occurrence matrix, gray level dependence matrix, gray level run length matrix, gray level size zone matrix, and neighboring gray tone difference matrix features. Feature normalization was then performed to reduce scale differences and to ensure compatibility with downstream unsupervised clustering. The feature categories, feature names, and extraction sources used in the public code pipeline are summarized in **Supplementary Table S1**.

Following the previously published ITH score framework and our related follow up studies, k means clustering was applied with a predefined cluster number of six, nc = 6. This parameter was specified as *a priori* to maintain methodological consistency with prior ITH based studies and to avoid dataset specific optimization of the clustering scheme (26, 27). The resulting clustered subregions were subsequently used to derive the ITH metrics. The 2D ITH score was calculated from the largest axial CT slice by integrating local radiomics features with global pixel distribution properties (21–25), whereas the 3D ITH score was generated from the entire tumor volume using the same feature set on volumetric CT, following the approach of Zuo et al. (26, 27).

#### 2D ITH score

The 2D ITH score quantifies intratumoral heterogeneity by integrating pixel wise local radiomics features with global pixel distribution information from the largest axial tumor slice.21–25 A 2 × 2 sliding window was used to extract 104 radiomics features for each pixel within the lesion. Unsupervised clustering then generated a categorical label map representing distinct intratumoral subregions, from which the 2D ITH score was computed using Equation 1:

(1) $$2\text{D ITH score}=1-\frac{1}{{\text{S}}_{\text{total}}}\sum_{\text{i}=1}^{nc}\frac{{\text{S}}_{\text{l},\text{max}}}{{\text{n}}_{l}}$$

where *n_c_* denotes the number of clusters, *n_l_* denotes the number of connected regions, *S_l,_*_max_ is the maximum area of each cluster, and *S_total_* is the total tumor area.

#### 3D ITH score

Using the methodology proposed by Zuo et al. (26, 27), the 3D ITH score assesses intratumoral heterogeneity across the entire nodule volume by extending the computational principles of the 2D analysis to volumetric CT data and incorporating topological optimization to improve spatial discrimination. The workflow begins with voxel wise retrieval of the same radiomics feature classes using a 2 × 2 × 2 sliding cubic window, which allows capture of anisotropic spatial characteristics that cannot be fully appreciated on two-dimensional slice-based analysis. To preserve spatial coherence, a 26-connectivity topology was adopted, incorporating face connected, edge connected, and corner connected relationships within the three-dimensional voxel lattice.

Volumetric clustering subsequently generated a 3D label map that uncovered infiltrative proliferation patterns that may be concealed when analysis relies only on a single slice, including improved identification of microlobular structures at the tumor invasive margin. From these spatially coherent subvolumes, the 3D ITH score was computed using the refined dispersion measure shown in Equation 2:

(2) $$3\text{D ITH score}=1-\frac{1}{{\text{V}}_{\text{total}}}\sum_{\text{l}=1}^{nc}\frac{{\text{V}}_{\text{l},\text{max}}}{{\text{m}}_{l}}$$

where *m_l_* denotes the number of connected volumes, V*_l,_*_max_ is the volume of each subregion, and *V*_total_ is the total tumor volume.

### Machine learning framework

#### Class-balancing methods

A marked class imbalance was observed in the dataset, with VPI positive cases accounting for only 13.3% to 13.4% of the cohort and VPI negative cases accounting for 86.6% to 86.7%. Such imbalance can bias the model toward the majority class, reduce sensitivity for the minority class, increase the risk of overfitting, and limit generalizability. To mitigate this issue, we applied the Synthetic Minority Oversampling Technique combined with Tomek link undersampling, SMOTETomek. This approach increases representation of the minority class by generating synthetic samples based on feature similarity and removes redundant or noisy samples from the majority class (28, 29). SMOTETomek was applied only within the training data during model development and was not applied to the external validation cohort.

#### Base machine learning framework

Six widely used supervised machine learning algorithms were selected as the base models for VPI prediction in LUAD: random forest (RF), adaptive boosting (AdaBoost), light gradient boosting machine (LightGBM), gradient boosting decision tree (GBDT), extreme gradient boosting (XGBoost), and categorical boosting (CatBoost). Each model offers distinct advantages: RF mitigates overfitting through bagging; AdaBoost iteratively reweights misclassified samples to enhance weak learners; LightGBM accelerates training via histogram-based optimization; GBDT sequentially minimizes loss gradients; XGBoost incorporates L1/L2 regularization for improved stability; and CatBoost automatically handles categorical variables to reduce prediction bias.

All input features underwent standardized preprocessing. Hyperparameters were optimized using 5-fold stratified cross-validation with grid search, and model selection was guided by maximizing the area under the curve (AUC), consistent with widely adopted practices in medical imaging classification (30–32).

#### Stacking ensemble machine learning framework

To improve prediction accuracy and robustness, a stacking ensemble framework was developed by integrating the six optimized base models, RF, AdaBoost, LightGBM, GBDT, XGBoost, and CatBoost. The stacking pipeline comprised three main stages. First, five-fold stratified cross validation was performed on the training set to generate out of fold predicted probabilities for each base model. Second, these six probability vectors were concatenated to form a meta feature matrix, which served as input to the meta classifier. Logistic regression was selected as the meta classifier because of its simplicity, interpretability, and ability to integrate linear combinations of meta features without unnecessary complexity.

Finally, the meta-classifier was trained using meta-features derived from the full training set. For external validation, CT data from the independent validation center were processed through the six base models to compute corresponding meta-features, which were subsequently entered into the trained meta-classifier to yield final VPI predictions. Hyperparameters of the meta-classifier were tuned using 5-fold cross-validation to reduce the risk of overfitting within the meta-feature space.

#### Model selection protocol

Following established methodologies (25–27, 30), the model selection process involved three sequential stages. First, the optimal model was identified based on the highest AUC achieved in the development process. Next, model interpretability was assessed using the SHAP framework, and all input features were ranked in descending order according to their absolute SHAP values.

Subsequently, a SHAP-guided iterative ablation analysis was conducted using a stepwise feature addition strategy. Beginning with the feature exhibiting the highest SHAP importance, a model was trained using 5-fold cross-validation, and mean AUC was evaluated on the external validation set. The feature with the second-highest SHAP value was then added, followed by repeated cross-validation and AUC assessment. This iterative process continued until all features were included. Throughout the procedure, dynamic trends in accumulated feature count and corresponding mean AUC were recorded and visualized using bar charts and line graphs. The iterative process was terminated once performance improvements plateaued, and the feature set at that point was designated as the most reliable subset for VPI prediction in LUAD.

### Comparative experiments

To examine the incremental value of integrating 2D and 3D ITH scores, as well as the performance of the proposed stacking framework, two additional comparative models were constructed and evaluated using the same training and external validation sets, with identical preprocessing procedures and evaluation metrics. (1) Clinicoradiologic model: this comparative model used the same stacking framework as the full model but excluded the 2D and 3D ITH scores and used only clinical and radiological variables, such as sex, age, nodule size, and CT density. (2) Radiomics signature: following conventional radiomics modeling paradigms (32–34), this model was built using 1,239 radiomics features and underwent a three step feature selection process: a univariate t test to remove features with P > 0.5, Pearson correlation analysis to eliminate redundant features with correlation coefficients greater than 0.9, and LASSO regression with 10 fold cross validation to identify the most predictive feature subset while minimizing overfitting (**Supplementary Figure 1**).

#### Statistical analysis

All statistical analyses were performed using R software, version 4.3.1, and Python, version 3.9.13. Continuous variables were reported as median with interquartile range, Q1 to Q3, and were compared using the Mann Whitney U test when normality assumptions were violated, or as mean ± standard deviation and compared using the independent samples t test when normality assumptions were met. Categorical variables were summarized as counts and percentages, and between group comparisons were performed using the chi square test. A two tailed P value of less than 0.05 was considered statistically significant. Model performance was primarily assessed by receiver operating characteristic analysis, with evaluation metrics including AUC, precision, recall, accuracy, and F1 score. Given the marked class imbalance, precision recall curves and the area under the precision recall curve were additionally calculated to complement ROC based assessment. For the primary study design, patients from Centers 1 and 2 were combined to form the development cohort, whereas Center 3 was reserved as the prespecified fixed external validation cohort. In addition, a supplementary leave one center out cross validation analysis was performed to assess cross center robustness, in which Centers 1 and 2 were sequentially held out as validation cohorts while the remaining centers served as the training cohort.

## Results

### Patient characteristics

A total of 1,301 patients with LUAD were enrolled from three independent centers, including 600 from Center 1, 310 from Center 2, and 391 from Center 3. For dataset partitioning, patients from Centers 1 and 2 were combined to form the training set (n = 910), while patients from Center 3 served as the external validation set (n = 391).

Clinicoradiological characteristics were recorded for all patients and compared between the training and external validation sets, as summarized in **Table 1**. Statistical analyses revealed no significant differences in these characteristics between the two sets (all p > 0.05), indicating a well-balanced cohort.

**Table 1:** Comparative analysis of clinicoradiographic features between the development and external validation sets.

Variables	Total (n = 1301)	Development set (n = 910)	External validation set (n = 391)	P value
Pathological diagnosis, n (%)				1
VPI-negative	1127 (86.6)	788 (86.6)	339 (86.7)	
VPI-positive	174 (13.4)	122 (13.4)	52 (13.3)	
Sex, n (%)				0.671
Female	849 (65.3)	590 (64.8)	259 (66.2)	
Male	452 (34.7)	320 (35.2)	132 (33.8)	
Age, y, Median (Q1, Q3)	57 (51, 66)	58 (51, 66)	56 (50, 65)	0.149
Nodule size, mm, Median (Q1, Q3)	17.9 (13.2, 24.5)	17.8 (13.2, 24.2)	18.3 (13.3, 25.1)	0.477
CT density, n (%)				0.385
pGGN	653 (50.2)	449 (49.3)	204 (52.2)	
SSN	326 (25.1)	226 (24.8)	100 (25.6)	
SN	322 (24.8)	235 (25.8)	87 (22.3)	
Location, n (%)				0.148
RUL	453 (34.8)	326 (35.8)	127 (32.5)	
RLL	101 (7.8)	78 (8.6)	23 (5.9)	
RML	226 (17.4)	149 (16.4)	77 (19.7)	
LUL	347 (26.7)	243 (26.7)	104 (26.6)	
LLL	174 (13.4)	114 (12.5)	60 (15.3)	
Boundary, n (%)				1
Well-defined	992 (76.2)	694 (76.3)	298 (76.2)	
Ill-defined	309 (23.8)	216 (23.7)	93 (23.8)	
Lobulation sign, n (%)				0.959
Absent	612 (47)	429 (47.1)	183 (46.8)	
Present	689 (53)	481 (52.9)	208 (53.2)	
Spiculation sign, n (%)				0.652
Absent	718 (55.2)	498 (54.7)	220 (56.3)	
Present	583 (44.8)	412 (45.3)	171 (43.7)	
Vascular convergence sign, n (%)				0.603
Absent	310 (23.8)	221 (24.3)	89 (22.8)	
Present	991 (76.2)	689 (75.7)	302 (77.2)	
Vacuole sign, n (%)				0.492
Absent	1072 (82.4)	745 (81.9)	327 (83.6)	
Present	229 (17.6)	165 (18.1)	64 (16.4)	
Pleural indentation sign, n (%)				0.904
Absent	574 (44.1)	400 (44)	174 (44.5)	
Present	727 (55.9)	510 (56)	217 (55.5)	
Shape, n (%)				0.741
Regular	738 (56.7)	513 (56.4)	225 (57.5)	
Irregular	563 (43.3)	397 (43.6)	166 (42.5)	
2D ITH score, Median (Q1, Q3)	0.5 (0.3, 0.7)	0.5 (0.3, 0.7)	0.5 (0.3, 0.7)	0.312
3D ITH score, Median (Q1, Q3)	0.8 (0.6, 0.9)	0.8 (0.6, 0.9)	0.8 (0.6, 0.9)	0.574

VPI, visceral pleural invasion; pGGN, pure ground glass nodule; SN, solid nodule; SSN, subsolid nodule; RUL, right upper lobe; RLL, right lower lobe; RML, right middle lobe; LUL, left upper lobe; LLL, left lower lobe; ITH, intratumoral heterogeneity.

### Base machine learning and stacking ensemble models

Six widely used supervised machine learning algorithms were employed as base classifiers for VPI prediction in LUAD: RF, GBDT, XGBoost, LightGBM, AdaBoost, and CatBoost. To further enhance predictive performance and robustness, a stacking ensemble classifier was constructed by integrating these six optimized base models.

**Figure 2:**
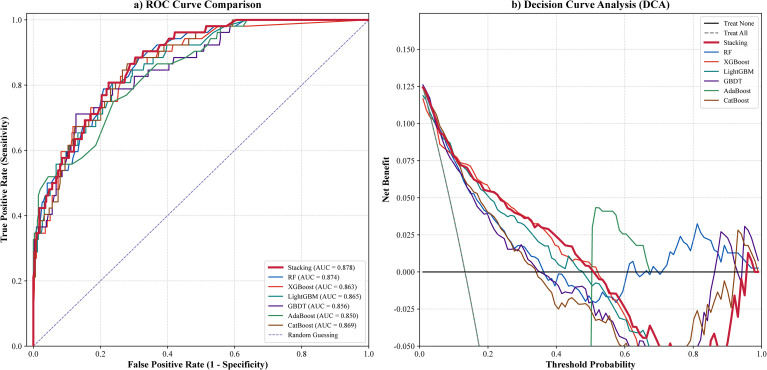
Receiver operating characteristic and decision curve analyses of the base machine learning models and the stacking ensemble classifier. **(A)** Receiver operating characteristic curves comparing the discriminative performance of six base models, RF, XGBoost, LightGBM, GBDT, AdaBoost, and CatBoost, and the stacking ensemble classifier. **(B)** Decision curve analysis showing the net clinical benefit of all models.

The diagnostic performance of all models was evaluated using ROC curve analysis and decision curve analysis, with results shown in **Figure 2A** and **Figure 2B**. As illustrated in **Figure 2A**, the stacking ensemble classifier achieved the highest AUC among all models, 0.878. **Figure 2B** further demonstrated the clinical utility of the stacking model. Within the threshold probability range in which VPI incidence in LUAD was below 50%, consistent with the observed prevalence of 13.3% to 13.4% in this study, the stacking classifier yielded the greatest net clinical benefit.

Detailed quantitative results are summarized in **Table 2**. Among all models, the stacking ensemble classifier achieved the highest AUC, whereas XGBoost remained highly competitive and showed slightly better performance on several threshold dependent metrics at the default operating point. In supplementary leave one center out analyses, discrimination performance remained relatively stable across centers; however, threshold dependent metrics such as recall and F1 score varied more substantially, indicating operating point sensitivity across institutions and the potential need for center specific threshold adjustment (**Supplementary Table 2**).

**Table 2:** Comparison of diagnostic performance between the base machine learning models and the stacking ensemble classifier.

Models	Accuracy	AUC	F1 Score	Precision	Recall
Stacking classifier	0.87	**0.878**	0.532	0.509	0.558
RF	0.849	0.874	0.487	0.444	0.538
XGBoost	0.872	0.865	0.554	0.517	0.596
LightGBM	0.862	0.865	0.534	0.484	0.596
GBDT	0.847	0.856	0.531	0.447	0.654
AdaBoost	0.806	0.85	0.441	0.357	0.577
CatBoost	0.841	0.869	0.53	0.438	0.673

RF, random forest; XGBoost, extreme gradient boosting; LightGBM, light gradient boosting machine; GBDT, gradient boosting decision tree; AdaBoost, adaptive boosting; AUC, area under the curve. Bold values indicate the highest value for each performance metric among the compared models.

To further evaluate performance under class imbalance, precision recall analysis was performed. The stacking model maintained robust discrimination, with a PR AUC of 0.627, which was substantially higher than the positive class prevalence of 0.133 in the test cohort (**Supplementary Figure 2**).

### Model interpretation for the stacking ensemble classifier

**Figure 3:**
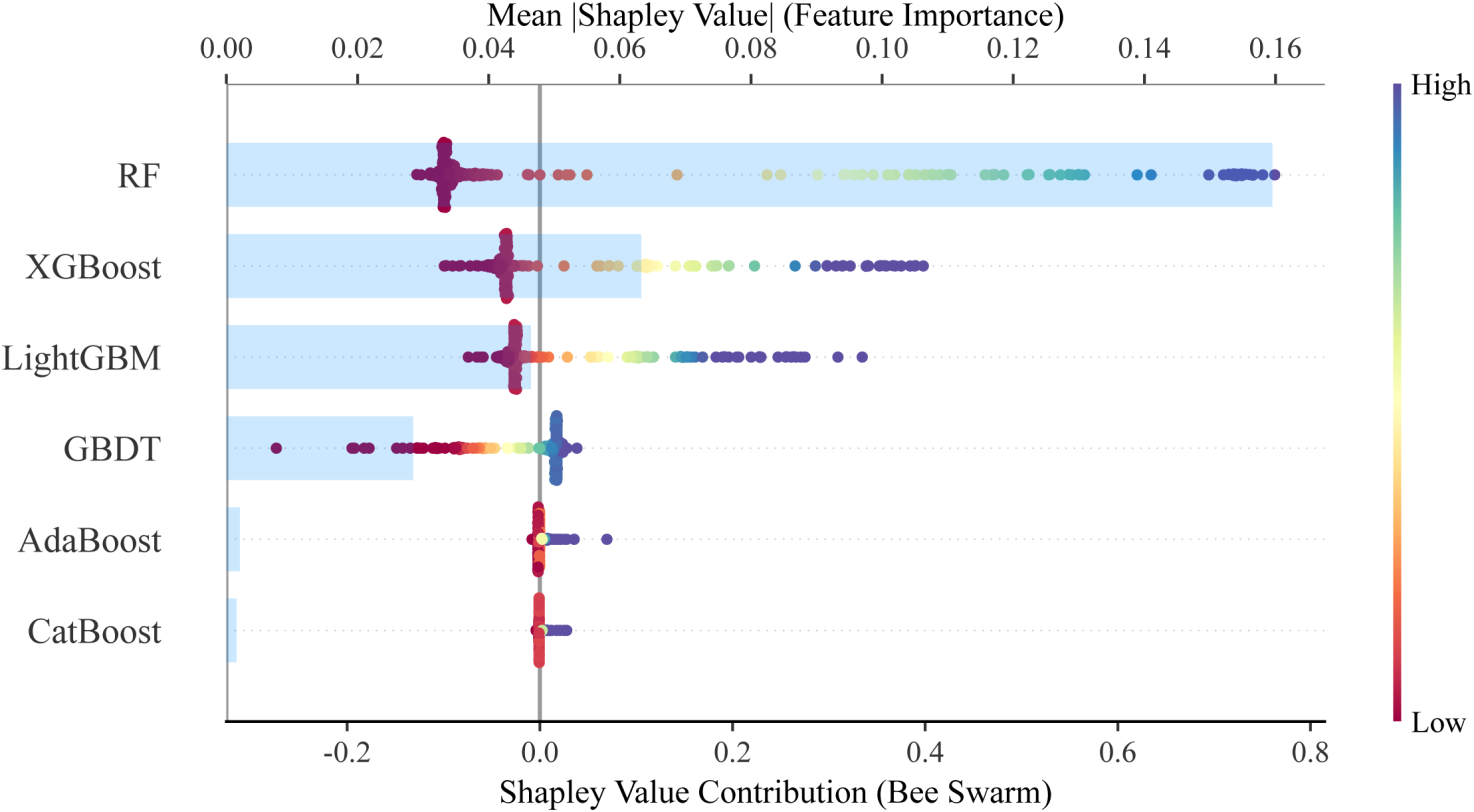
SHAP-based analysis of the contribution of six base classifiers to the stacking ensemble classifier. This image quantifies and visualizes the relative contribution of each base model (RF, XGBoost, LightGBM, GBDT, AdaBoost, CatBoost) to the final prediction performance of the stacking ensemble classifier, reflecting the complementary strengths of different algorithms.

**Figure 4:**
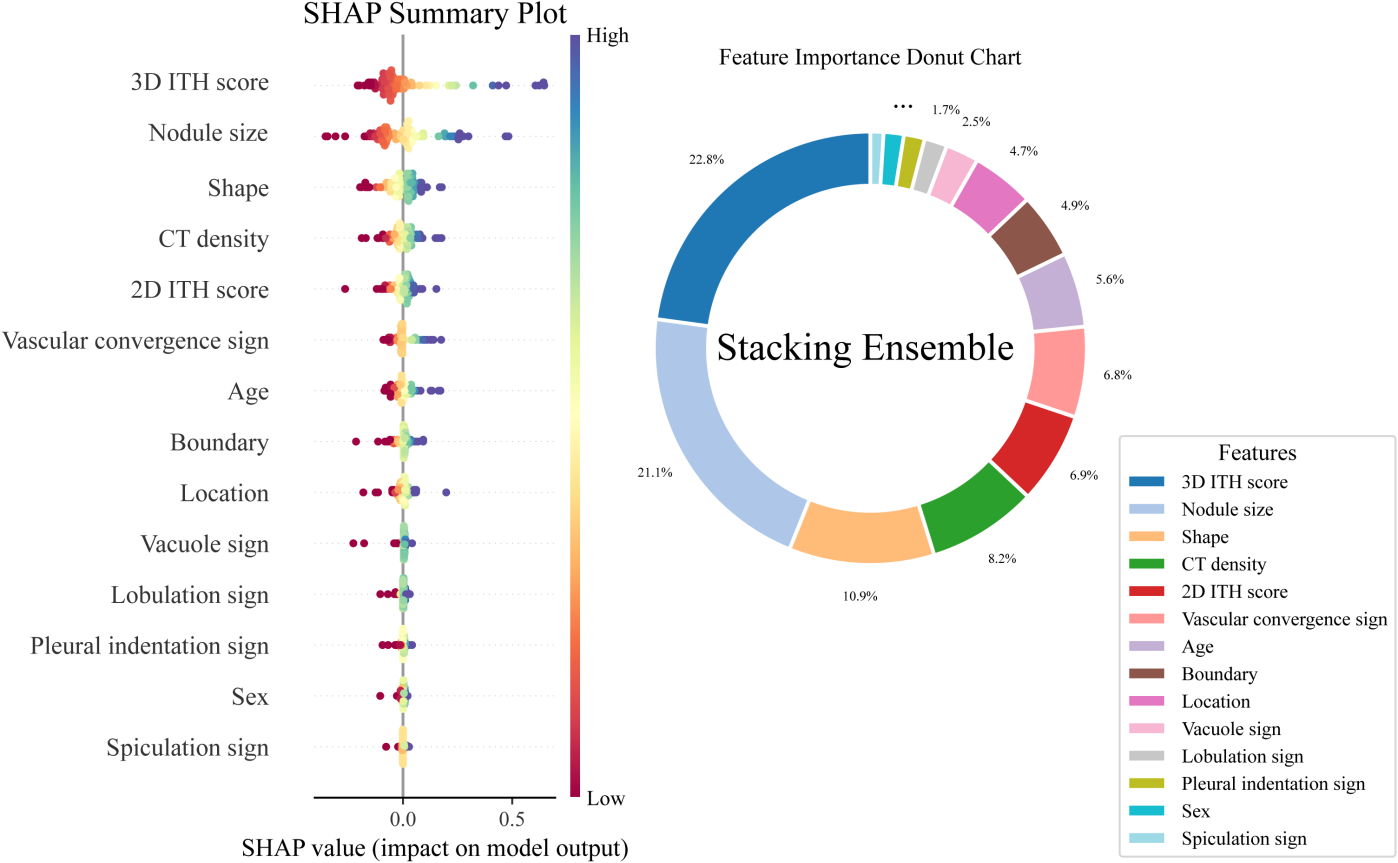
Interpretation of the stacking ensemble classifier using the SHAP framework. This image illustrates the model’s prediction logic, including SHAP summary plots that show feature importance and the direction of each feature’s influence on VPI prediction.

The SHAP framework was used to interpret the stacking ensemble classifier. SHAP analysis of the six base classifiers revealed that the RF contributed most to the ensemble (**Figure 3**). Evaluation of the overall stacking model showed that the 3D ITH score was the most influential feature, accounting for 22.8% of the predictive contribution (**Figure 4**).

**Figure 5:**
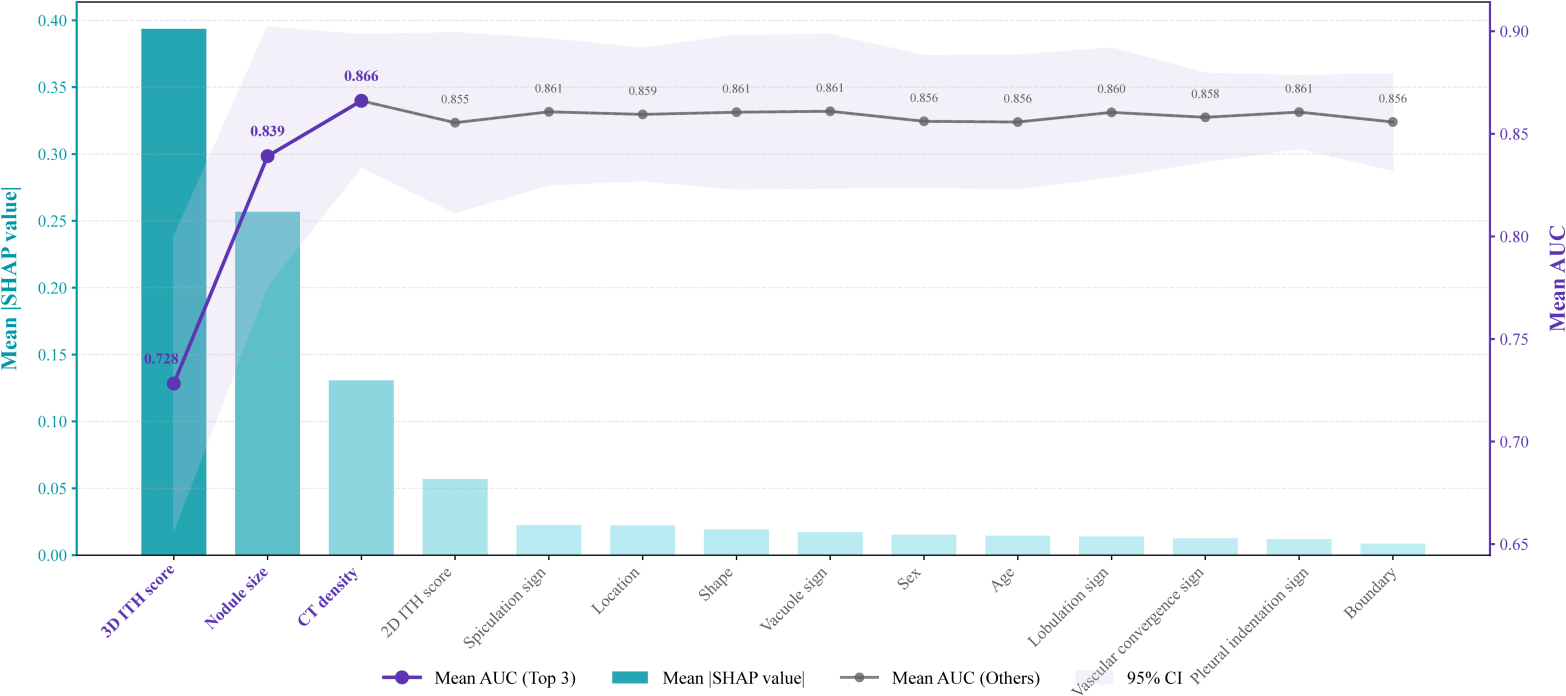
SHAP-guided ablation experiments. This image presents the results of iterative feature ablation analysis, evaluating the changes in model performance (AUC) after sequentially removing features ranked by SHAP importance, to identify the most critical predictors for VPI prediction.

To further assess feature robustness, SHAP-based ablation experiments were performed (**Figure 5**). These results indicated that the model’s AUC increased steadily when the top three features were included but declined when additional features were added. Consequently, the 3D ITH score, nodule size, and CT density were identified as the most critical features for maintaining the predictive performance of the stacking ensemble classifier.

### Comparative experiments

**Figure 6:**
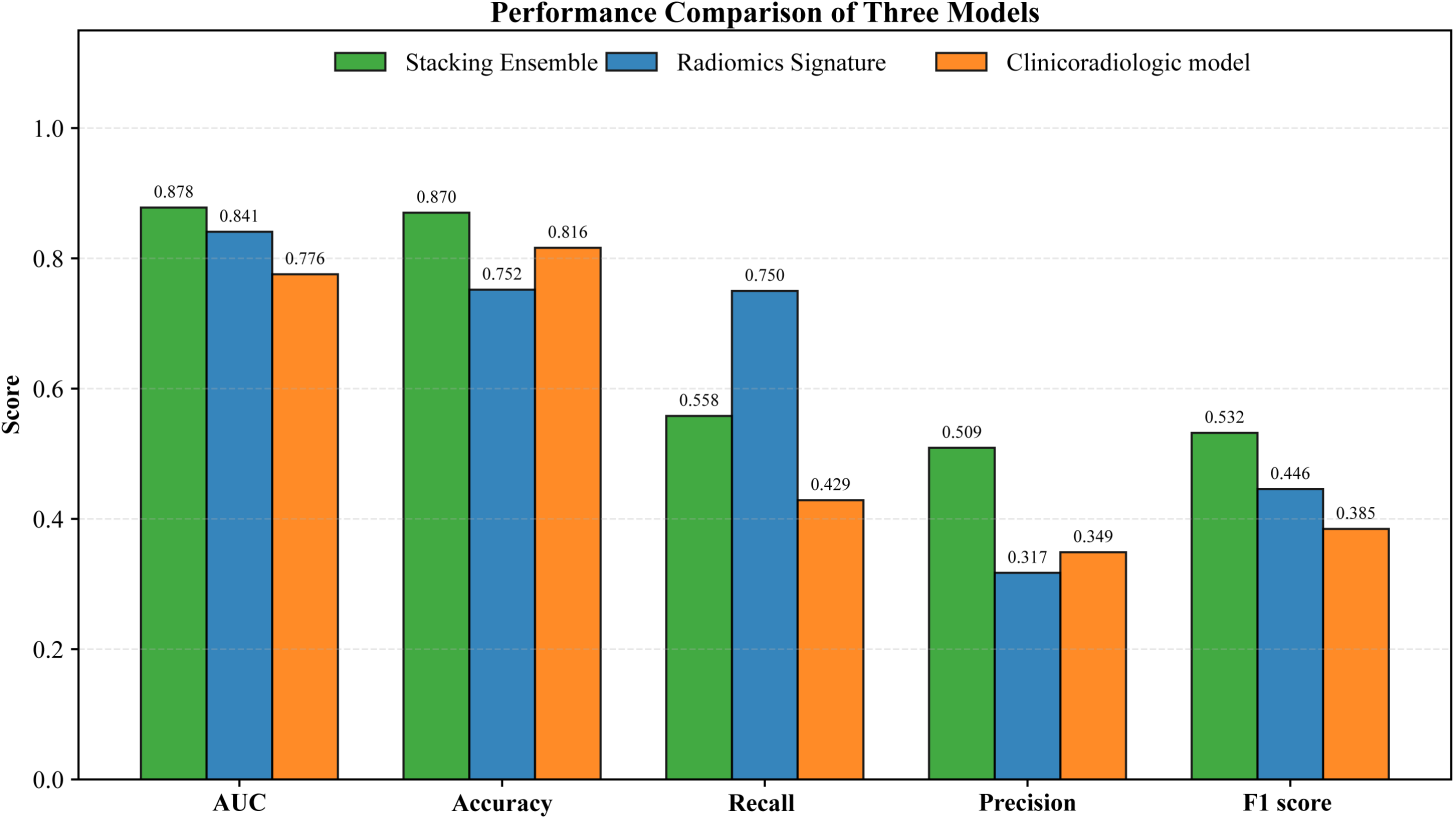
Bar charts comparing the diagnostic performance of the stacking ensemble classifier, radiomics signature, and clinicoradiologic model in comparative experiments. The charts display key performance metrics (AUC, accuracy, precision, recall, F1 score) of the three models, clearly demonstrating the superior comprehensive performance of the stacking ensemble classifier.

To further examine comparative performance, two additional models were evaluated, and the results are summarized in **Table 3** and **Figure 6**. The clinicoradiologic comparative model was trained using the same stacking framework as the full model but used only clinical and radiological variables as input features, and it achieved an AUC of 0.776. The radiomics signature was constructed using a conventional radiomics workflow. A total of 1,239 radiomics features were initially extracted and then filtered through a three step procedure consisting of univariate t testing, Pearson correlation analysis, and LASSO regression with 10 fold cross validation, yielding 9 final features and an AUC of 0.841 (**Supplementary Figure 3** and **4**).

**Table 3:** Comparative analysis of diagnostic performance among the clinicoradiologic model, radiomics signature, and stacking ensemble model.

Models	Accuracy	AUC	F1 Score	Precision	Recall
Stacking ensemble model	0.87	**0.878**	0.532	0.509	0.558
Radiomics signature	0.752	0.841	0.446	0.317	0.75
Clinicoradiologic model	0.816	0.776	0.385	0.349	0.429

AUC, area under the curve. Bold values indicate the highest value for each performance metric among the compared models.

The stacking ensemble classifier achieved the highest AUC among the three models and showed the most favorable overall balance between discrimination and classification performance for VPI prediction in LUAD.

## Discussion

To our knowledge, this multicenter large scale study is the first to integrate 3D ITH scores with clinicoradiologic features using machine learning for preoperative prediction of VPI in LUAD. The stacking ensemble classifier achieved the highest AUC and favorable decision curve performance, whereas XGBoost remained a strong single model comparator. SHAP analysis identified the 3D ITH score as the most influential predictor, highlighting its central role in capturing VPI related biological heterogeneity.

Traditional radiomics approaches generally assume a uniform tumor composition during volumetric analysis. This assumption may obscure critical intratumoral heterogeneity and biologically relevant subregions (14–17). To address this limitation, Li et al. (21) proposed a 2D ITH score based on CT imaging. By integrating local pixel level information with global image features, this score can characterize tumor heterogeneity more precisely. The 2D ITH metric has shown value in evaluating biological characteristics of LUAD (21–25) and has also been applied to other malignancies, including breast cancer (35, 36), colorectal cancer (37), hepatocellular carcinoma (38), and gastric cancer (39), often outperforming conventional radiomics approaches. Nevertheless, an important limitation of the 2D ITH score is its dependence on the largest tumor cross sectional slice (21–25, 35–39), which does not fully capture heterogeneity across the entire tumor volume.

More recently, Zuo et al. (26, 27) developed a 3D ITH score derived from the entire tumor volume by integrating local radiomic descriptors with global pixel distribution patterns from volumetric CT images. This volumetric approach enables a more comprehensive assessment of tumor biology in LUAD. Consistent with this concept, SHAP analysis in the present study showed that the 3D ITH score was the most influential predictor of model performance. SHAP guided ablation further identified the 3D ITH score, nodule size, and CT density as the most important features for preserving predictive accuracy, whereas the 2D ITH score contributed less substantially. Collectively, these findings support the capacity of the 3D ITH score to capture intratumoral heterogeneity across multiple dimensions and to provide a more faithful representation of the complex tumor landscape.

From a clinical perspective, the predicted probability generated by the model should be interpreted as a preoperative risk estimate rather than a definitive treatment rule. Low predicted probabilities may support routine preoperative management, whereas intermediate probabilities may warrant closer integration of pleural imaging signs, clinical judgment, and multidisciplinary discussion. High predicted probabilities may increase suspicion for VPI and support more cautious surgical planning. Nevertheless, because no prospective threshold validation study was performed, these probabilities should be used as an adjunct to clinical and radiological assessment rather than as the sole basis for determining surgical extent. Our findings also confirmed CT density and nodule size as additional predictors of VPI, consistent with previous studies highlighting radiological features related to tumor morphology and density as useful indicators of tumor invasiveness (10–12, 40–42).

Several limitations should be acknowledged. First, the retrospective study design may have introduced selection bias, as inclusion depended on complete clinical and imaging data, and inter center variation in diagnostic or treatment protocols may have affected cohort homogeneity. Second, the lack of long term follow up limited evaluation of the model’s value for guiding surgical decisions and its association with recurrence, overall survival, or disease-free survival. Third, the 2D and 3D ITH scores relied solely on CT derived features without genomic or molecular integration, which limited insight into the biological mechanisms linking intratumoral heterogeneity to VPI. Fourth, no user-friendly clinical tool is currently available for implementation, which limits real world applicability. Fifth, although discrimination performance remained relatively robust across centers, the observed variability in threshold dependent metrics suggests that direct deployment may require local recalibration or threshold customization according to center specific prevalence, workflow, and clinical objectives. Finally, although SMOTETomek improved minority class learning, it may have affected probability calibration. Future studies should compare resampling-based strategies with cost sensitive learning and should incorporate dedicated calibration analyses.

## Conclusions

The model integrating 3D ITH scores with clinicoradiologic features represents a practical advance in preoperative prediction of VPI in LUAD. With prospective validation and development of user-friendly tools, this approach may contribute to more personalized oncologic care.

## Data Availability

The datasets generated and/or analyzed during the current study are not publicly available because they contain potentially sensitive patient information, but are available from the corresponding author on reasonable request and subject to institutional and ethical approval.

## Supplementary material

Supplementary Figure 1 LASSO regression with 10-fold cross validation for radiomics feature selection. This figure shows the process used to identify the most predictive radiomics feature subset while minimizing overfitting risk, including the lambda selection plot and coefficient path plot.

Supplementary Figure 2 Precision recall curve of the stacking ensemble classifier on the test set. The solid line represents the precision recall curve for the stacking model, which achieved a PR AUC of 0.627. The dashed gray line indicates the no skill baseline, corresponding to the prevalence of the positive class in the test cohort, 0.133.

Supplementary Figure 3 Correlation coefficient heatmap of the final radiomics signature. The heatmap visualizes pairwise correlations among the 9 selected radiomics features and confirms the absence of severe multicollinearity after feature screening.

Supplementary Figure 4 Lollipop plot of the final radiomics signature. The plot displays the relative importance or coefficient values of the 9 selected radiomics features and illustrates the contribution of each feature to the radiomics signature.

## References

[1] Siegel RL Miller KD Jemal A . Cancer statistics, 2023. CA Cancer J Clin. (2023) 73:17–48. doi: 10.3322/caac.21763. PMID: 36633525

[2] Zuo Z Zhang G Song P Yang J Li S Zhong Z . Survival nomogram for stage IB non-small-cell lung cancer patients, based on the SEER database and an external validation cohort. Ann Surg Oncol. (2021) 28:3941–50. doi: 10.1245/s10434-020-09362-0. PMID: 33249521

[3] De Giglio A Di Federico A Gelsomino F Ardizzoni A . Prognostic relevance of pleural invasion for resected NSCLC patients undergoing adjuvant treatments: a propensity score-matched analysis of SEER database. Lung Cancer. (2021) 161:18–25. doi: 10.1016/j.lungcan.2021.08.017. PMID: 34492552

[4] Qi M Bian D Zhang J Zhu X Zhou C Zhang L . The modification of T description according to visceral pleural invasion and tumor size from 3.1 cm to 4.0 cm in non-small cell lung cancer: a retrospective analysis based on the SEER database. Lung Cancer. (2021) 158:47–54. doi: 10.1016/j.lungcan.2021.06.003. PMID: 34119932

[5] Wang S Zhang B Qian J Qiao R Xu J Zhang L . Proposal on incorporating lymphovascular invasion as a T-descriptor for stage I lung cancer. Lung Cancer. (2018) 125:245–52. doi: 10.1016/j.lungcan.2018.09.024. PMID: 30429028

[6] Goldstraw P Chansky K Crowley J Rami-Porta R Asamura H Eberhardt WEE . The IASLC lung cancer staging project: proposals for revision of the TNM stage groupings in the forthcoming (eighth) edition of the TNM classification for lung cancer. J Thorac Oncol. (2016) 11:39–51. doi: 10.1016/j.jtho.2015.09.009. PMID: 26762738

[7] Yu Y Huang R Wang P Wang S Ling X Zhang P . Sublobectomy versus lobectomy for long-term survival outcomes of early-stage non-small cell lung cancer with a tumor size ≤2 cm accompanied by visceral pleural invasion: a SEER population-based study. J Thorac Dis. (2020) 12:592–604. doi: 10.21037/jtd.2019.12.121. PMID: 32274125 PMC7138986

[8] Wang Y Lyu D Fan L Liu S . Research progress in predicting visceral pleural invasion of lung cancer: a narrative review. Transl Cancer Res. (2024) 13:462–70. doi: 10.21037/tcr-23-1318. PMID: 38410233 PMC10894335

[9] Huang Y Zhao B Yan R Zhang C Geng Z Mei P . AI-based prediction of pathological risk factors in lung adenocarcinoma from CT imaging: bridging innovation and clinical practice. Front Oncol. (2025) 15:1687360. doi: 10.3389/fonc.2025.1687360. PMID: 41323376 PMC12658906

[10] Zhang L Zhao L Lin X Zhao S Pan W Wang D . Comparison of tumor non-specific and PD-L1 specific imaging by near-infrared fluorescence/Cerenkov luminescence dual-modality in-situ imaging. Mol Imaging. (2024) 23:15353508241261473. doi: 10.1177/15353508241261473. PMID: 38952401 PMC11208884

[11] Archibald SJ Holland JP Korde A Martins AF Shuhendler AJ Scott PJH . Combining nuclear medicine with other modalities: future prospect for multimodality imaging. Mol Imaging. (2024) 23:15353508241245265. doi: 10.1177/15353508241245265. PMID: 38952398 PMC11208883

[12] Chen S Ren S Guo K Daniels MJ Wang Z Chen R . Preoperative differentiation of serous cystic neoplasms from mucin-producing pancreatic cystic neoplasms using a CT-based radiomics nomogram. Abdom Radiol (NY). (2021) 46:2637–46. doi: 10.1007/s00261-021-02954-8. PMID: 33558952

[13] Ren S Qin B Daniels MJ Zeng L Tian Y Wang ZQ . Developing and validating a computed tomography radiomics strategy to predict lymph node metastasis in pancreatic cancer. World J Radiol. (2025) 17:109373. doi: 10.4329/wjr.v17.i8.109373. PMID: 40901350 PMC12400248

[14] Cui WJ Wang C Jia L Ren S Duan SF Cui C . Differentiation between G1 and G2/G3 phyllodes tumors of breast using mammography and mammographic texture analysis. Front Oncol. (2019) 9:433. doi: 10.3389/fonc.2019.00433. PMID: 31192133 PMC6548862

[15] Lyu D Wang Y Tu W Hu S Ma Y Huang W . Prediction of visceral pleural invasion of clinical stage IA lung adenocarcinoma based on computed tomography features. Transl Cancer Res. (2025) 14:1596–608. doi: 10.21037/tcr-24-2015. PMID: 40224965 PMC11985210

[16] Benchoufi M Matzner-Lober E Molinari N Jannot AS Soyer P . Interobserver agreement issues in radiology. Diagn Interv Imaging. (2020) 101:639–41. doi: 10.1016/j.diii.2020.09.001. PMID: 32958434

[17] Zhao J Wang T Wang B Satishkumar BM Ding L Sun X . Deep learning radiomics fusion model to predict visceral pleural invasion of clinical stage IA lung adenocarcinoma: a multicenter study. J Cardiothorac Surg. (2025) 20:246. doi: 10.1186/s13019-025-03488-6. PMID: 40437608 PMC12121141

[18] Chen M Copley SJ Viola P Lu H Aboagye EO . Radiomics and artificial intelligence for precision medicine in lung cancer treatment. Semin Cancer Biol. (2023) 93:97–113. doi: 10.1016/j.semcancer.2023.05.004. PMID: 37211292

[19] Zuo Z Deng J Ge W Zhou Y Liu H Zhang W . Quantifying intratumoral heterogeneity within sub-regions to predict high-grade patterns in clinical stage I solid lung adenocarcinoma. BMC Cancer. (2025) 25:51. doi: 10.1186/s12885-025-13445-0. PMID: 39789523 PMC11720805

[20] Zuo Z Zhang G Lin S Xue Q Qi W Zhang W . Radiomics nomogram based on optimal volume of interest derived from high-resolution CT for preoperative prediction of IASLC grading in clinical IA lung adenocarcinomas: a multi-center, large-population study. Technol Cancer Res Treat. (2024) 23:15330338241300734. doi: 10.1177/15330338241300734. PMID: 39569528 PMC11580084

[21] Li J Qiu Z Zhang C Chen S Wang M Meng Q . ITHscore: comprehensive quantification of intra-tumor heterogeneity in NSCLC by multi-scale radiomic features. Eur Radiol. (2022) 33:893–903. doi: 10.1007/s00330-022-09055-0. PMID: 36001124

[22] Zhang J Sha J Liu W Zhou Y Liu H Zuo Z . Quantification of intratumoral heterogeneity: distinguishing histological subtypes in clinical T1 stage lung adenocarcinoma presenting as pure ground-glass nodules on computed tomography. Acad Radiol. (2024) 31:4244–55. doi: 10.1016/j.acra.2024.04.008. PMID: 38627129

[23] Zheng H Chen W Qi W Liu H Zuo Z . Enhancing the prediction of the invasiveness of pulmonary adenocarcinomas presenting as pure ground-glass nodules: integrating intratumor heterogeneity score with clinical-radiological features via machine learning in a multicenter study. Digit Health. (2024) 10:1–12. doi: 10.1177/20552076241289181. PMID: 39381817 PMC11459516

[24] Qi H Zuo Z Lin S Chen Y Li H Hu D . Assessment of intratumor heterogeneity for preoperatively predicting the invasiveness of pulmonary adenocarcinomas manifesting as pure ground-glass nodules. Quant Imaging Med Surg. (2024) 15:272–86. doi: 10.21037/qims-24-734. PMID: 39839051 PMC11744125

[25] Zuo Z Zeng Y Deng J Lin S Qi W Fan X . Intratumoral heterogeneity score enhances invasiveness prediction in pulmonary ground-glass nodules via stacking ensemble machine learning. Insights Imaging. (2025) 16:209. doi: 10.1186/s13244-025-02097-0. PMID: 41006794 PMC12474818

[26] Zuo Z Fan X Zeng Y Qi W Liu W Zhang J . Multiperspective tumor heterogeneity metrics for preoperative prediction of IASLC grading in clinical stage IA lung adenocarcinomas: a multicenter study. Comput Methods Programs BioMed. (2025) 274:109137. doi: 10.1016/j.cmpb.2025.109137. PMID: 41197251

[27] Zuo Z Fan X Zeng Y Qi W Liu W Li W . Topologically distinct 2D and 3D intratumoral heterogeneity scores for preoperatively predicting invasiveness in stage I lung adenocarcinoma: a multicenter study. PloS Digit Health. (2026) 5:e0001246. doi: 10.1371/journal.pdig.0001246. PMID: 41719325 PMC12923145

[28] Li Z Kamnitsas K Glocker B . Analyzing overfitting under class imbalance in neural networks for image segmentation. IEEE Trans Med Imaging. (2021) 40:1065–77. doi: 10.1109/tmi.2020.3046692. PMID: 33351758

[29] Chen S Deng T Yang Q Li J Shen J Luo X . Development and validation of an explainable machine learning model for predicting postoperative pulmonary complications after lung cancer surgery: a machine learning study. EClinicalMedicine. (2025) 86:103386. doi: 10.1016/j.eclinm.2025.103386. PMID: 40791887 PMC12337024

[30] Zhang M Zheng Y Maidaiti X Liang B Wei Y Sun F . Integrating machine learning into statistical methods in disease risk prediction modeling: a systematic review. Health Data Sci. (2024) 4:165. doi: 10.34133/hds.0165. PMID: 39050273 PMC11266123

[31] Ling T Zuo Z Huang M Ma J Wu L . Stacking classifiers based on integrated machine learning model: fusion of CT radiomics and clinical biomarkers to predict lymph node metastasis in locally advanced gastric cancer patients after neoadjuvant chemotherapy. BMC Cancer. (2025) 25:834. doi: 10.1186/s12885-025-14259-w. PMID: 40329193 PMC12057267

[32] Li Y Ding J Wu K Qi W Lin S Chen G . Ensemble machine learning classifiers combining CT radiomics and clinical-radiological features for preoperative prediction of pathological invasiveness in lung adenocarcinoma presenting as part-solid nodules: a multicenter retrospective study. Technol Cancer Res Treat. (2025) 24:15330338251351365. doi: 10.1177/15330338251351365. PMID: 40525253 PMC12174711

[33] Yang X Fan X Lin S Zhou Y Liu H Wang X . Assessment of lymphovascular invasion in breast cancer using a combined MRI morphological features, radiomics, and deep learning approach based on dynamic contrast-enhanced MRI. J Magn Reson Imaging. (2024) 59:2238–49. doi: 10.1002/jmri.29060. PMID: 37855421

[34] Zuo Z Zhang G Chen J Xue Q Lin S Zeng Y . CT radiomic nomogram using optimal volume of interest for preoperatively predicting invasive mucinous adenocarcinomas in patients with incidental pulmonary nodules: a multicenter, large-scale study. Technol Cancer Res Treat. (2024) 23:15330338241308307. doi: 10.1177/15330338241308307. PMID: 39703067 PMC11662315

[35] Huang Y Wang X Cao Y Lan X Hu X Mou F . Nomogram for predicting neoadjuvant chemotherapy response in breast cancer using MRI-based intratumoral heterogeneity quantification. Radiology. (2025) 315:e241805. doi: 10.1148/radiol.241805. PMID: 40232145

[36] Zuo Z Feng Y Deng J Yang X Zeng Y Fan X . Dynamic contrast-enhanced MRI-derived intratumoral heterogeneity quantification score: improving lymphovascular invasion and invasive breast cancer recurrence-free survival predictions. Radiography. (2026) 32:103204. doi: 10.1016/j.radi.2025.103204. PMID: 41566494

[37] Zhou Y Zuo Z Zhao J Tan Y Deng J Wei X . Development and validation of time-to-event machine learning models for predicting disease-free survival in patients with locally advanced colorectal cancer: a multicenter cohort study. Ann Surg Oncol. (2026) 33:1288–300. doi: 10.1245/s10434-025-18815-3 41329308

[38] Zhao J Zhou M Tan Y Wei X Jiang W Tong L . Preoperative CT-based topologically distinct intratumoral heterogeneity scores for predicting intratumoral tertiary lymphoid structures and outcomes in hepatocellular carcinoma: a multicenter study. Eur J Surg Oncol. (2026) 52:111756. doi: 10.1016/j.ejso.2026.111756 41880709

[39] Li J Li Z Wang Y Li Y Zhang J Li Z . CT radiomics-based intratumoral and intertumoral heterogeneity indicators for prognosis prediction in gastric cancer patients receiving neoadjuvant chemotherapy. Eur Radiol. (2025) 35:4448–60. doi: 10.1007/s00330-025-11430-6 39953151

[40] Onoda H Higashi M Murakami T Tao H Yokoyama S Kunihiro Y . Correlation between pleural tags on CT and visceral pleural invasion of peripheral lung cancer that does not appear touching the pleural surface. Eur Radiol. (2021) 31:9022–9. doi: 10.1007/s00330-021-07869-y. PMID: 34019129

[41] Kim H Goo JM Kim YT Park CM . CT-defined visceral pleural invasion in T1 lung adenocarcinoma: lack of relationship to disease-free survival. Radiology. (2019) 292:741–9. doi: 10.1148/radiol.2019190297. PMID: 31361207

[42] Hsu JS Han IT Tsai TH Lin SF Jaw TS Liu GC . Pleural tags on CT scans to predict visceral pleural invasion of non-small cell lung cancer that does not abut the pleura. Radiology. (2016) 279:590–6. doi: 10.1148/radiol.2015151120. PMID: 26653684

