# Supplementary figures and images for: 3D intratumoral heterogeneity-based quantitative score from chest CT for preoperative prediction of visceral pleural invasion in lung adenocarcinoma: a multicenter study
Source: Front Oncol. 2026 May 12;16:1837845. doi: 10.3389/fonc.2026.1837845 (PMC13201124; doi:10.3389/fonc.2026.1837845)

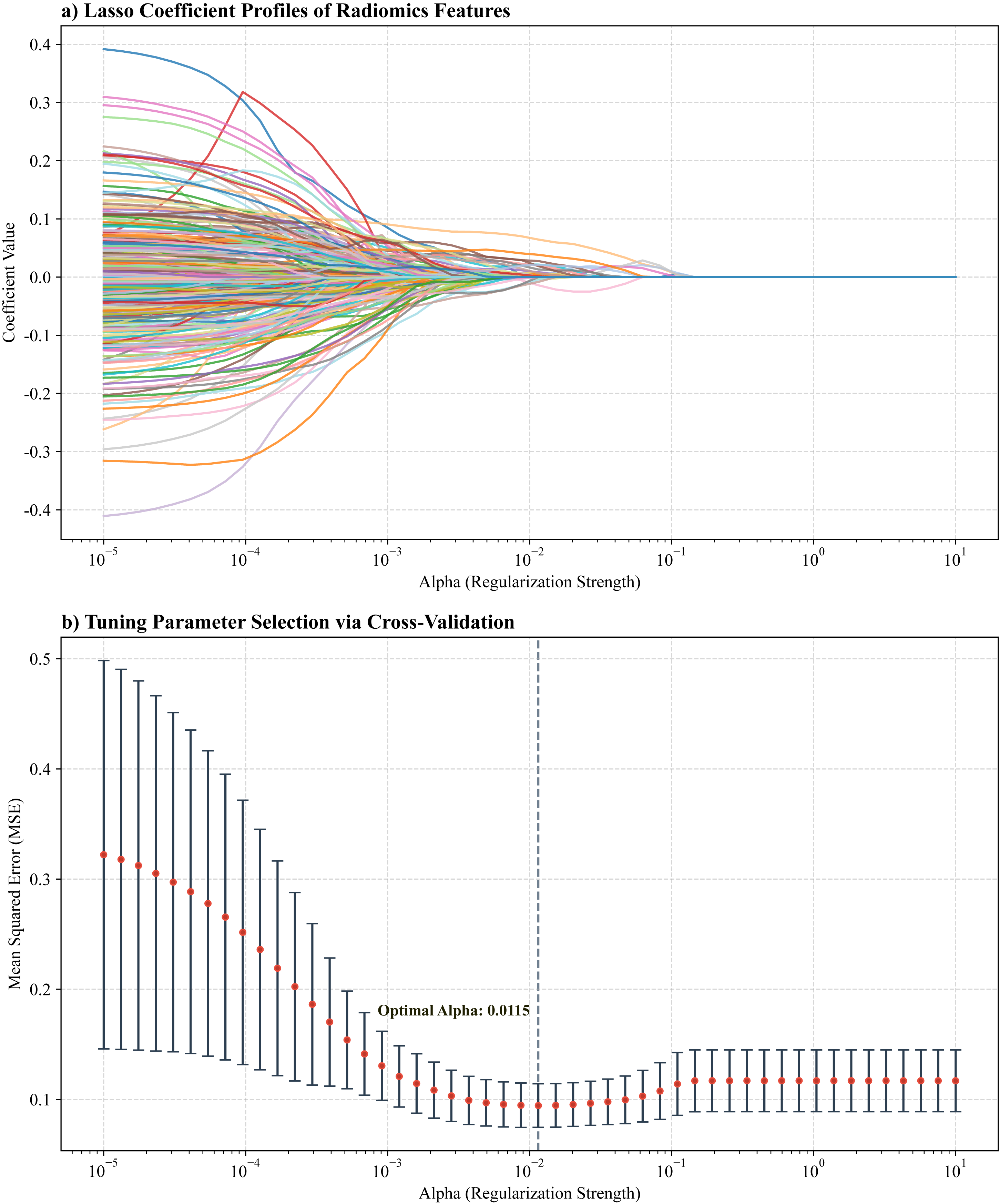

Supplement: Supplementary Figure 1 — LASSO regression with 10-fold cross validation for radiomics feature selection. This figure shows the process used to identify the most predictive radiomics feature subset while minimizing overfitting risk, including the lambda selection plot and coefficient path plot. [file Image1.tif]

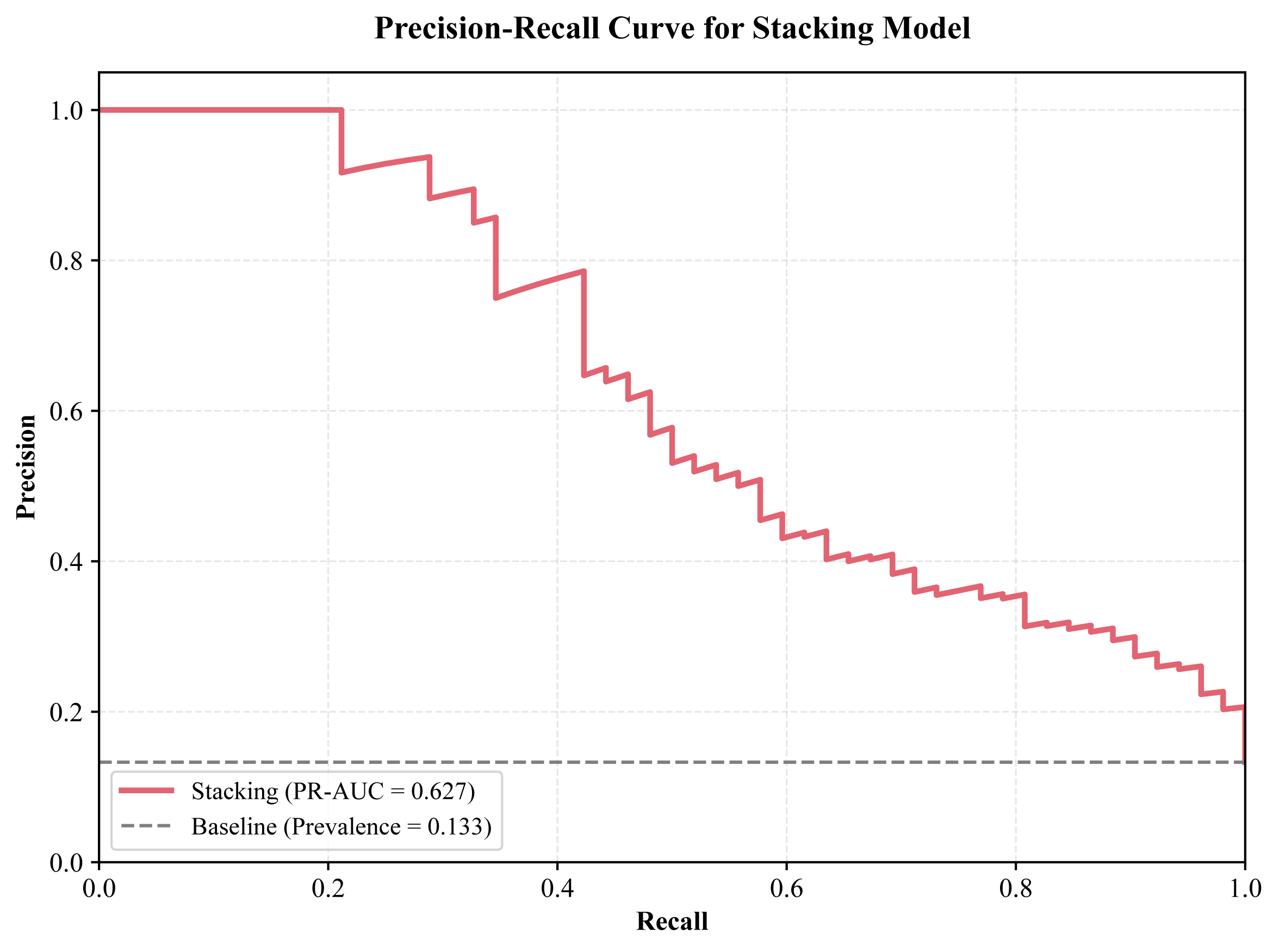

Supplement: Supplementary Figure 2 — Precision recall curve of the stacking ensemble classifier on the test set. The solid line represents the precision recall curve for the stacking model, which achieved a PR AUC of 0.627. The dashed gray line indicates the no skill baseline, corresponding to the prevalence of the positive class in the test cohort, 0.133. [file Image2.tif]

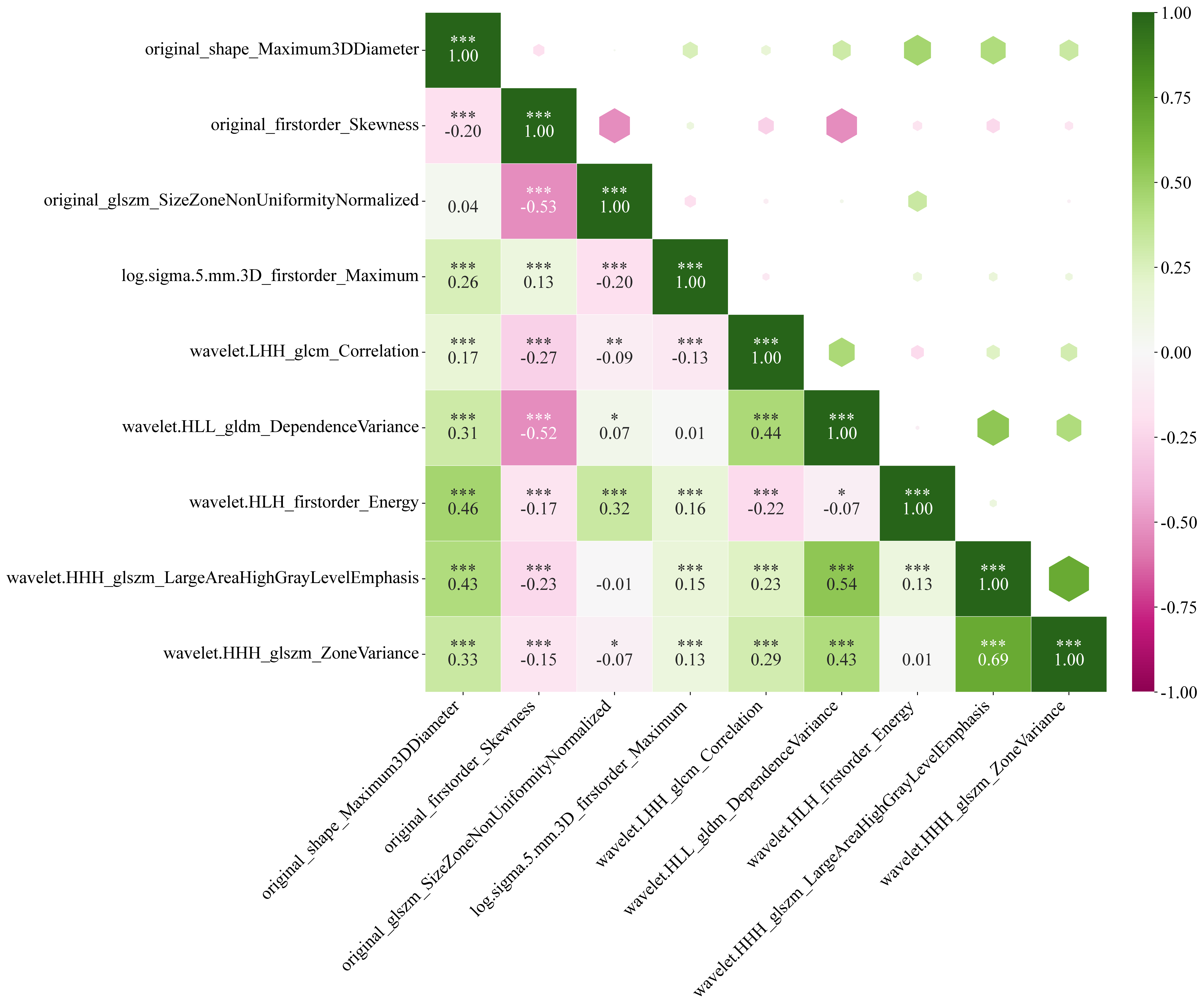

Supplement: Supplementary Figure 3 — Correlation coefficient heatmap of the final radiomics signature. The heatmap visualizes pairwise correlations among the 9 selected radiomics features and confirms the absence of severe multicollinearity after feature screening. [file Image3.tif]

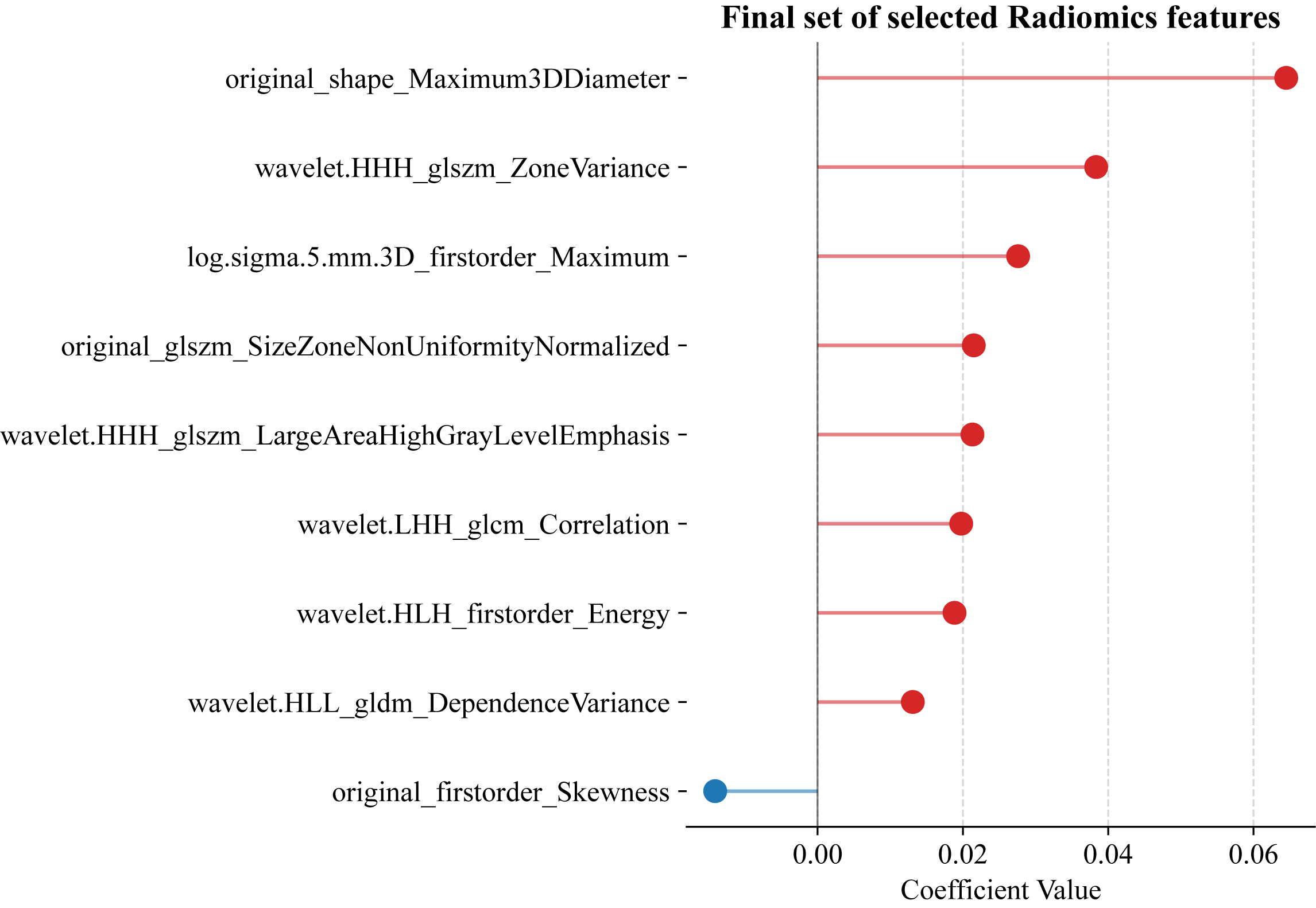

Supplement: Supplementary Figure 4 — Lollipop plot of the final radiomics signature. The plot displays the relative importance or coefficient values of the 9 selected radiomics features and illustrates the contribution of each feature to the radiomics signature. [file Image4.tif]
