# Supplementary material for: 3D intratumoral heterogeneity-based quantitative score from chest CT for preoperative prediction of visceral pleural invasion in lung adenocarcinoma: a multicenter study
Source: Front Oncol. 2026 May 12;16:1837845. doi: 10.3389/fonc.2026.1837845 (PMC13201124; doi:10.3389/fonc.2026.1837845)
Supplement: Supplementary file 5 [file Table1.docx]

**Supplementary Table S1. Radiomics feature categories and feature names in the public ITHscore code pipeline**

| No. | Feature class | Feature name | Extraction source | Source URL | Notes |
| --- | --- | --- | --- | --- | --- |
| 1 | First-order | Energy | PyRadiomics original feature class | https://pyradiomics.readthedocs.io/ | Public ITHscore code uses RadiomicsFeatureExtractor() in the feature extraction pipeline. |
| 2 | First-order | TotalEnergy | PyRadiomics original feature class | https://pyradiomics.readthedocs.io/ | Public ITHscore code uses RadiomicsFeatureExtractor() in the feature extraction pipeline. |
| 3 | First-order | Entropy | PyRadiomics original feature class | https://pyradiomics.readthedocs.io/ | Public ITHscore code uses RadiomicsFeatureExtractor() in the feature extraction pipeline. |
| 4 | First-order | Minimum | PyRadiomics original feature class | https://pyradiomics.readthedocs.io/ | Public ITHscore code uses RadiomicsFeatureExtractor() in the feature extraction pipeline. |
| 5 | First-order | 10Percentile | PyRadiomics original feature class | https://pyradiomics.readthedocs.io/ | Public ITHscore code uses RadiomicsFeatureExtractor() in the feature extraction pipeline. |
| 6 | First-order | 90Percentile | PyRadiomics original feature class | https://pyradiomics.readthedocs.io/ | Public ITHscore code uses RadiomicsFeatureExtractor() in the feature extraction pipeline. |
| 7 | First-order | Maximum | PyRadiomics original feature class | https://pyradiomics.readthedocs.io/ | Public ITHscore code uses RadiomicsFeatureExtractor() in the feature extraction pipeline. |
| 8 | First-order | Mean | PyRadiomics original feature class | https://pyradiomics.readthedocs.io/ | Public ITHscore code uses RadiomicsFeatureExtractor() in the feature extraction pipeline. |
| 9 | First-order | Median | PyRadiomics original feature class | https://pyradiomics.readthedocs.io/ | Public ITHscore code uses RadiomicsFeatureExtractor() in the feature extraction pipeline. |
| 10 | First-order | InterquartileRange | PyRadiomics original feature class | https://pyradiomics.readthedocs.io/ | Public ITHscore code uses RadiomicsFeatureExtractor() in the feature extraction pipeline. |
| 11 | First-order | Range | PyRadiomics original feature class | https://pyradiomics.readthedocs.io/ | Public ITHscore code uses RadiomicsFeatureExtractor() in the feature extraction pipeline. |
| 12 | First-order | MeanAbsoluteDeviation | PyRadiomics original feature class | https://pyradiomics.readthedocs.io/ | Public ITHscore code uses RadiomicsFeatureExtractor() in the feature extraction pipeline. |
| 13 | First-order | RobustMeanAbsoluteDeviation | PyRadiomics original feature class | https://pyradiomics.readthedocs.io/ | Public ITHscore code uses RadiomicsFeatureExtractor() in the feature extraction pipeline. |
| 14 | First-order | RootMeanSquared | PyRadiomics original feature class | https://pyradiomics.readthedocs.io/ | Public ITHscore code uses RadiomicsFeatureExtractor() in the feature extraction pipeline. |
| 15 | First-order | Skewness | PyRadiomics original feature class | https://pyradiomics.readthedocs.io/ | Public ITHscore code uses RadiomicsFeatureExtractor() in the feature extraction pipeline. |
| 16 | First-order | Kurtosis | PyRadiomics original feature class | https://pyradiomics.readthedocs.io/ | Public ITHscore code uses RadiomicsFeatureExtractor() in the feature extraction pipeline. |
| 17 | First-order | Variance | PyRadiomics original feature class | https://pyradiomics.readthedocs.io/ | Public ITHscore code uses RadiomicsFeatureExtractor() in the feature extraction pipeline. |
| 18 | First-order | Uniformity | PyRadiomics original feature class | https://pyradiomics.readthedocs.io/ | Public ITHscore code uses RadiomicsFeatureExtractor() in the feature extraction pipeline. |
| 19 | Shape (3D) | MeshVolume | PyRadiomics original feature class | https://pyradiomics.readthedocs.io/ | Public ITHscore code uses RadiomicsFeatureExtractor() in the feature extraction pipeline. |
| 20 | Shape (3D) | VoxelVolume | PyRadiomics original feature class | https://pyradiomics.readthedocs.io/ | Public ITHscore code uses RadiomicsFeatureExtractor() in the feature extraction pipeline. |
| 21 | Shape (3D) | SurfaceArea | PyRadiomics original feature class | https://pyradiomics.readthedocs.io/ | Public ITHscore code uses RadiomicsFeatureExtractor() in the feature extraction pipeline. |
| 22 | Shape (3D) | SurfaceVolumeRatio | PyRadiomics original feature class | https://pyradiomics.readthedocs.io/ | Public ITHscore code uses RadiomicsFeatureExtractor() in the feature extraction pipeline. |
| 23 | Shape (3D) | Sphericity | PyRadiomics original feature class | https://pyradiomics.readthedocs.io/ | Public ITHscore code uses RadiomicsFeatureExtractor() in the feature extraction pipeline. |
| 24 | Shape (3D) | Maximum3DDiameter | PyRadiomics original feature class | https://pyradiomics.readthedocs.io/ | Public ITHscore code uses RadiomicsFeatureExtractor() in the feature extraction pipeline. |
| 25 | Shape (3D) | Maximum2DDiameterSlice | PyRadiomics original feature class | https://pyradiomics.readthedocs.io/ | Public ITHscore code uses RadiomicsFeatureExtractor() in the feature extraction pipeline. |
| 26 | Shape (3D) | Maximum2DDiameterColumn | PyRadiomics original feature class | https://pyradiomics.readthedocs.io/ | Public ITHscore code uses RadiomicsFeatureExtractor() in the feature extraction pipeline. |
| 27 | Shape (3D) | Maximum2DDiameterRow | PyRadiomics original feature class | https://pyradiomics.readthedocs.io/ | Public ITHscore code uses RadiomicsFeatureExtractor() in the feature extraction pipeline. |
| 28 | Shape (3D) | MajorAxisLength | PyRadiomics original feature class | https://pyradiomics.readthedocs.io/ | Public ITHscore code uses RadiomicsFeatureExtractor() in the feature extraction pipeline. |
| 29 | Shape (3D) | MinorAxisLength | PyRadiomics original feature class | https://pyradiomics.readthedocs.io/ | Public ITHscore code uses RadiomicsFeatureExtractor() in the feature extraction pipeline. |
| 30 | Shape (3D) | LeastAxisLength | PyRadiomics original feature class | https://pyradiomics.readthedocs.io/ | Public ITHscore code uses RadiomicsFeatureExtractor() in the feature extraction pipeline. |
| 31 | Shape (3D) | Elongation | PyRadiomics original feature class | https://pyradiomics.readthedocs.io/ | Public ITHscore code uses RadiomicsFeatureExtractor() in the feature extraction pipeline. |
| 32 | Shape (3D) | Flatness | PyRadiomics original feature class | https://pyradiomics.readthedocs.io/ | Public ITHscore code uses RadiomicsFeatureExtractor() in the feature extraction pipeline. |
| 33 | GLCM | Autocorrelation | PyRadiomics original feature class | https://pyradiomics.readthedocs.io/ | Public ITHscore code uses RadiomicsFeatureExtractor() in the feature extraction pipeline. |
| 34 | GLCM | JointAverage | PyRadiomics original feature class | https://pyradiomics.readthedocs.io/ | Public ITHscore code uses RadiomicsFeatureExtractor() in the feature extraction pipeline. |
| 35 | GLCM | ClusterProminence | PyRadiomics original feature class | https://pyradiomics.readthedocs.io/ | Public ITHscore code uses RadiomicsFeatureExtractor() in the feature extraction pipeline. |
| 36 | GLCM | ClusterShade | PyRadiomics original feature class | https://pyradiomics.readthedocs.io/ | Public ITHscore code uses RadiomicsFeatureExtractor() in the feature extraction pipeline. |
| 37 | GLCM | ClusterTendency | PyRadiomics original feature class | https://pyradiomics.readthedocs.io/ | Public ITHscore code uses RadiomicsFeatureExtractor() in the feature extraction pipeline. |
| 38 | GLCM | Contrast | PyRadiomics original feature class | https://pyradiomics.readthedocs.io/ | Public ITHscore code uses RadiomicsFeatureExtractor() in the feature extraction pipeline. |
| 39 | GLCM | Correlation | PyRadiomics original feature class | https://pyradiomics.readthedocs.io/ | Public ITHscore code uses RadiomicsFeatureExtractor() in the feature extraction pipeline. |
| 40 | GLCM | DifferenceAverage | PyRadiomics original feature class | https://pyradiomics.readthedocs.io/ | Public ITHscore code uses RadiomicsFeatureExtractor() in the feature extraction pipeline. |
| 41 | GLCM | DifferenceEntropy | PyRadiomics original feature class | https://pyradiomics.readthedocs.io/ | Public ITHscore code uses RadiomicsFeatureExtractor() in the feature extraction pipeline. |
| 42 | GLCM | DifferenceVariance | PyRadiomics original feature class | https://pyradiomics.readthedocs.io/ | Public ITHscore code uses RadiomicsFeatureExtractor() in the feature extraction pipeline. |
| 43 | GLCM | JointEnergy | PyRadiomics original feature class | https://pyradiomics.readthedocs.io/ | Public ITHscore code uses RadiomicsFeatureExtractor() in the feature extraction pipeline. |
| 44 | GLCM | JointEntropy | PyRadiomics original feature class | https://pyradiomics.readthedocs.io/ | Public ITHscore code uses RadiomicsFeatureExtractor() in the feature extraction pipeline. |
| 45 | GLCM | Imc1 | PyRadiomics original feature class | https://pyradiomics.readthedocs.io/ | Public ITHscore code uses RadiomicsFeatureExtractor() in the feature extraction pipeline. |
| 46 | GLCM | Imc2 | PyRadiomics original feature class | https://pyradiomics.readthedocs.io/ | Public ITHscore code uses RadiomicsFeatureExtractor() in the feature extraction pipeline. |
| 47 | GLCM | Idm | PyRadiomics original feature class | https://pyradiomics.readthedocs.io/ | Public ITHscore code uses RadiomicsFeatureExtractor() in the feature extraction pipeline. |
| 48 | GLCM | MCC | PyRadiomics original feature class | https://pyradiomics.readthedocs.io/ | Public ITHscore code uses RadiomicsFeatureExtractor() in the feature extraction pipeline. |
| 49 | GLCM | Idmn | PyRadiomics original feature class | https://pyradiomics.readthedocs.io/ | Public ITHscore code uses RadiomicsFeatureExtractor() in the feature extraction pipeline. |
| 50 | GLCM | Id | PyRadiomics original feature class | https://pyradiomics.readthedocs.io/ | Public ITHscore code uses RadiomicsFeatureExtractor() in the feature extraction pipeline. |
| 51 | GLCM | Idn | PyRadiomics original feature class | https://pyradiomics.readthedocs.io/ | Public ITHscore code uses RadiomicsFeatureExtractor() in the feature extraction pipeline. |
| 52 | GLCM | InverseVariance | PyRadiomics original feature class | https://pyradiomics.readthedocs.io/ | Public ITHscore code uses RadiomicsFeatureExtractor() in the feature extraction pipeline. |
| 53 | GLCM | MaximumProbability | PyRadiomics original feature class | https://pyradiomics.readthedocs.io/ | Public ITHscore code uses RadiomicsFeatureExtractor() in the feature extraction pipeline. |
| 54 | GLCM | SumAverage | PyRadiomics original feature class | https://pyradiomics.readthedocs.io/ | Public ITHscore code uses RadiomicsFeatureExtractor() in the feature extraction pipeline. |
| 55 | GLCM | SumEntropy | PyRadiomics original feature class | https://pyradiomics.readthedocs.io/ | Public ITHscore code uses RadiomicsFeatureExtractor() in the feature extraction pipeline. |
| 56 | GLCM | SumSquares | PyRadiomics original feature class | https://pyradiomics.readthedocs.io/ | Public ITHscore code uses RadiomicsFeatureExtractor() in the feature extraction pipeline. |
| 57 | GLDM | SmallDependenceEmphasis | PyRadiomics original feature class | https://pyradiomics.readthedocs.io/ | Public ITHscore code uses RadiomicsFeatureExtractor() in the feature extraction pipeline. |
| 58 | GLDM | LargeDependenceEmphasis | PyRadiomics original feature class | https://pyradiomics.readthedocs.io/ | Public ITHscore code uses RadiomicsFeatureExtractor() in the feature extraction pipeline. |
| 59 | GLDM | GrayLevelNonUniformity | PyRadiomics original feature class | https://pyradiomics.readthedocs.io/ | Public ITHscore code uses RadiomicsFeatureExtractor() in the feature extraction pipeline. |
| 60 | GLDM | DependenceNonUniformity | PyRadiomics original feature class | https://pyradiomics.readthedocs.io/ | Public ITHscore code uses RadiomicsFeatureExtractor() in the feature extraction pipeline. |
| 61 | GLDM | DependenceNonUniformityNormalized | PyRadiomics original feature class | https://pyradiomics.readthedocs.io/ | Public ITHscore code uses RadiomicsFeatureExtractor() in the feature extraction pipeline. |
| 62 | GLDM | GrayLevelVariance | PyRadiomics original feature class | https://pyradiomics.readthedocs.io/ | Public ITHscore code uses RadiomicsFeatureExtractor() in the feature extraction pipeline. |
| 63 | GLDM | DependenceVariance | PyRadiomics original feature class | https://pyradiomics.readthedocs.io/ | Public ITHscore code uses RadiomicsFeatureExtractor() in the feature extraction pipeline. |
| 64 | GLDM | DependenceEntropy | PyRadiomics original feature class | https://pyradiomics.readthedocs.io/ | Public ITHscore code uses RadiomicsFeatureExtractor() in the feature extraction pipeline. |
| 65 | GLDM | LowGrayLevelEmphasis | PyRadiomics original feature class | https://pyradiomics.readthedocs.io/ | Public ITHscore code uses RadiomicsFeatureExtractor() in the feature extraction pipeline. |
| 66 | GLDM | HighGrayLevelEmphasis | PyRadiomics original feature class | https://pyradiomics.readthedocs.io/ | Public ITHscore code uses RadiomicsFeatureExtractor() in the feature extraction pipeline. |
| 67 | GLDM | SmallDependenceLowGrayLevelEmphasis | PyRadiomics original feature class | https://pyradiomics.readthedocs.io/ | Public ITHscore code uses RadiomicsFeatureExtractor() in the feature extraction pipeline. |
| 68 | GLDM | SmallDependenceHighGrayLevelEmphasis | PyRadiomics original feature class | https://pyradiomics.readthedocs.io/ | Public ITHscore code uses RadiomicsFeatureExtractor() in the feature extraction pipeline. |
| 69 | GLDM | LargeDependenceLowGrayLevelEmphasis | PyRadiomics original feature class | https://pyradiomics.readthedocs.io/ | Public ITHscore code uses RadiomicsFeatureExtractor() in the feature extraction pipeline. |
| 70 | GLDM | LargeDependenceHighGrayLevelEmphasis | PyRadiomics original feature class | https://pyradiomics.readthedocs.io/ | Public ITHscore code uses RadiomicsFeatureExtractor() in the feature extraction pipeline. |
| 71 | GLRLM | ShortRunEmphasis | PyRadiomics original feature class | https://pyradiomics.readthedocs.io/ | Public ITHscore code uses RadiomicsFeatureExtractor() in the feature extraction pipeline. |
| 72 | GLRLM | LongRunEmphasis | PyRadiomics original feature class | https://pyradiomics.readthedocs.io/ | Public ITHscore code uses RadiomicsFeatureExtractor() in the feature extraction pipeline. |
| 73 | GLRLM | GrayLevelNonUniformity | PyRadiomics original feature class | https://pyradiomics.readthedocs.io/ | Public ITHscore code uses RadiomicsFeatureExtractor() in the feature extraction pipeline. |
| 74 | GLRLM | GrayLevelNonUniformityNormalized | PyRadiomics original feature class | https://pyradiomics.readthedocs.io/ | Public ITHscore code uses RadiomicsFeatureExtractor() in the feature extraction pipeline. |
| 75 | GLRLM | RunLengthNonUniformity | PyRadiomics original feature class | https://pyradiomics.readthedocs.io/ | Public ITHscore code uses RadiomicsFeatureExtractor() in the feature extraction pipeline. |
| 76 | GLRLM | RunLengthNonUniformityNormalized | PyRadiomics original feature class | https://pyradiomics.readthedocs.io/ | Public ITHscore code uses RadiomicsFeatureExtractor() in the feature extraction pipeline. |
| 77 | GLRLM | RunPercentage | PyRadiomics original feature class | https://pyradiomics.readthedocs.io/ | Public ITHscore code uses RadiomicsFeatureExtractor() in the feature extraction pipeline. |
| 78 | GLRLM | GrayLevelVariance | PyRadiomics original feature class | https://pyradiomics.readthedocs.io/ | Public ITHscore code uses RadiomicsFeatureExtractor() in the feature extraction pipeline. |
| 79 | GLRLM | RunVariance | PyRadiomics original feature class | https://pyradiomics.readthedocs.io/ | Public ITHscore code uses RadiomicsFeatureExtractor() in the feature extraction pipeline. |
| 80 | GLRLM | RunEntropy | PyRadiomics original feature class | https://pyradiomics.readthedocs.io/ | Public ITHscore code uses RadiomicsFeatureExtractor() in the feature extraction pipeline. |
| 81 | GLRLM | LowGrayLevelRunEmphasis | PyRadiomics original feature class | https://pyradiomics.readthedocs.io/ | Public ITHscore code uses RadiomicsFeatureExtractor() in the feature extraction pipeline. |
| 82 | GLRLM | HighGrayLevelRunEmphasis | PyRadiomics original feature class | https://pyradiomics.readthedocs.io/ | Public ITHscore code uses RadiomicsFeatureExtractor() in the feature extraction pipeline. |
| 83 | GLRLM | ShortRunLowGrayLevelEmphasis | PyRadiomics original feature class | https://pyradiomics.readthedocs.io/ | Public ITHscore code uses RadiomicsFeatureExtractor() in the feature extraction pipeline. |
| 84 | GLRLM | ShortRunHighGrayLevelEmphasis | PyRadiomics original feature class | https://pyradiomics.readthedocs.io/ | Public ITHscore code uses RadiomicsFeatureExtractor() in the feature extraction pipeline. |
| 85 | GLRLM | LongRunLowGrayLevelEmphasis | PyRadiomics original feature class | https://pyradiomics.readthedocs.io/ | Public ITHscore code uses RadiomicsFeatureExtractor() in the feature extraction pipeline. |
| 86 | GLRLM | LongRunHighGrayLevelEmphasis | PyRadiomics original feature class | https://pyradiomics.readthedocs.io/ | Public ITHscore code uses RadiomicsFeatureExtractor() in the feature extraction pipeline. |
| 87 | GLSZM | SmallAreaEmphasis | PyRadiomics original feature class | https://pyradiomics.readthedocs.io/ | Public ITHscore code uses RadiomicsFeatureExtractor() in the feature extraction pipeline. |
| 88 | GLSZM | LargeAreaEmphasis | PyRadiomics original feature class | https://pyradiomics.readthedocs.io/ | Public ITHscore code uses RadiomicsFeatureExtractor() in the feature extraction pipeline. |
| 89 | GLSZM | GrayLevelNonUniformity | PyRadiomics original feature class | https://pyradiomics.readthedocs.io/ | Public ITHscore code uses RadiomicsFeatureExtractor() in the feature extraction pipeline. |
| 90 | GLSZM | GrayLevelNonUniformityNormalized | PyRadiomics original feature class | https://pyradiomics.readthedocs.io/ | Public ITHscore code uses RadiomicsFeatureExtractor() in the feature extraction pipeline. |
| 91 | GLSZM | SizeZoneNonUniformity | PyRadiomics original feature class | https://pyradiomics.readthedocs.io/ | Public ITHscore code uses RadiomicsFeatureExtractor() in the feature extraction pipeline. |
| 92 | GLSZM | SizeZoneNonUniformityNormalized | PyRadiomics original feature class | https://pyradiomics.readthedocs.io/ | Public ITHscore code uses RadiomicsFeatureExtractor() in the feature extraction pipeline. |
| 93 | GLSZM | ZonePercentage | PyRadiomics original feature class | https://pyradiomics.readthedocs.io/ | Public ITHscore code uses RadiomicsFeatureExtractor() in the feature extraction pipeline. |
| 94 | GLSZM | GrayLevelVariance | PyRadiomics original feature class | https://pyradiomics.readthedocs.io/ | Public ITHscore code uses RadiomicsFeatureExtractor() in the feature extraction pipeline. |
| 95 | GLSZM | ZoneVariance | PyRadiomics original feature class | https://pyradiomics.readthedocs.io/ | Public ITHscore code uses RadiomicsFeatureExtractor() in the feature extraction pipeline. |
| 96 | GLSZM | ZoneEntropy | PyRadiomics original feature class | https://pyradiomics.readthedocs.io/ | Public ITHscore code uses RadiomicsFeatureExtractor() in the feature extraction pipeline. |
| 97 | GLSZM | LowGrayLevelZoneEmphasis | PyRadiomics original feature class | https://pyradiomics.readthedocs.io/ | Public ITHscore code uses RadiomicsFeatureExtractor() in the feature extraction pipeline. |
| 98 | GLSZM | HighGrayLevelZoneEmphasis | PyRadiomics original feature class | https://pyradiomics.readthedocs.io/ | Public ITHscore code uses RadiomicsFeatureExtractor() in the feature extraction pipeline. |
| 99 | GLSZM | SmallAreaLowGrayLevelEmphasis | PyRadiomics original feature class | https://pyradiomics.readthedocs.io/ | Public ITHscore code uses RadiomicsFeatureExtractor() in the feature extraction pipeline. |
| 100 | GLSZM | SmallAreaHighGrayLevelEmphasis | PyRadiomics original feature class | https://pyradiomics.readthedocs.io/ | Public ITHscore code uses RadiomicsFeatureExtractor() in the feature extraction pipeline. |
| 101 | GLSZM | LargeAreaLowGrayLevelEmphasis | PyRadiomics original feature class | https://pyradiomics.readthedocs.io/ | Public ITHscore code uses RadiomicsFeatureExtractor() in the feature extraction pipeline. |
| 102 | GLSZM | LargeAreaHighGrayLevelEmphasis | PyRadiomics original feature class | https://pyradiomics.readthedocs.io/ | Public ITHscore code uses RadiomicsFeatureExtractor() in the feature extraction pipeline. |
| 103 | NGTDM | Coarseness | PyRadiomics original feature class | https://pyradiomics.readthedocs.io/ | Public ITHscore code uses RadiomicsFeatureExtractor() in the feature extraction pipeline. |
| 104 | NGTDM | Contrast | PyRadiomics original feature class | https://pyradiomics.readthedocs.io/ | Public ITHscore code uses RadiomicsFeatureExtractor() in the feature extraction pipeline. |
| 105 | NGTDM | Busyness | PyRadiomics original feature class | https://pyradiomics.readthedocs.io/ | Public ITHscore code uses RadiomicsFeatureExtractor() in the feature extraction pipeline. |
| 106 | NGTDM | Complexity | PyRadiomics original feature class | https://pyradiomics.readthedocs.io/ | Public ITHscore code uses RadiomicsFeatureExtractor() in the feature extraction pipeline. |
| 107 | NGTDM | Strength | PyRadiomics original feature class | https://pyradiomics.readthedocs.io/ | Public ITHscore code uses RadiomicsFeatureExtractor() in the feature extraction pipeline. |
