# Supplementary material for: 3D intratumoral heterogeneity-based quantitative score from chest CT for preoperative prediction of visceral pleural invasion in lung adenocarcinoma: a multicenter study
Source: Front Oncol. 2026 May 12;16:1837845. doi: 10.3389/fonc.2026.1837845 (PMC13201124; doi:10.3389/fonc.2026.1837845)
Supplement: Supplementary file 6 [file Table2.docx]

| **Supplementary Table 2.  External Validation Performance of the Stacking Ensemble Classifier Across Different Centers** | | | | | | | |
| --- | --- | --- | --- | --- | --- | --- | --- |
| External Validation Set | Sample size | Training Set | Accuracy | AUC | F1 score | Precision | Recall |
| Center 3 | 391 | Center 1+Center 2 | 0.87 | 0.878 | 0.532 | 0.509 | 0.558 |
| Center 1 | 600 | Center 2+Center 3 | 0.792 | 0.89 | 0.806 | 0.812 | 0.8 |
| Center 2 | 310 | Center 1+Center 3 | 0.787 | 0.872 | 0.787 | 0.792 | 0.782 |
| ***Abbreviation:*** AUC, area under the curve. | | | | | | | |
